# Autism Research: An Objective Quantitative Review of Progress and Focus Between 1994 and 2015

**DOI:** 10.3389/fpsyg.2018.01526

**Published:** 2018-08-23

**Authors:** Caroline P. Whyatt, Elizabeth B. Torres

**Affiliations:** Psychology, Rutgers University – The State University of New Jersey–Busch Campus, Piscataway, NJ, United States

**Keywords:** autism, quantitative review, graph theory, connectivity metrics, bibliometrics

## Abstract

The nosology and epidemiology of Autism has undergone transformation following consolidation of once disparate disorders under the umbrella diagnostic, autism spectrum disorders. Despite this re-conceptualization, research initiatives, including the NIMH’s Research Domain Criteria and Precision Medicine, highlight the need to bridge psychiatric and psychological classification methodologies with biomedical techniques. Combining traditional bibliometric co-word techniques, with tenets of graph theory and network analysis, this article provides an objective thematic review of research between 1994 and 2015 to consider evolution and focus. Results illustrate growth in Autism research since 2006, with nascent focus on physiology. However, modularity and citation analytics demonstrate dominance of subjective psychological or psychiatric constructs, which may impede progress in the identification and stratification of biomarkers as endorsed by new research initiatives.

“*There are in fact two things, science and opinion; the former begets knowledge, the latter ignorance*”

-Hippocrates.

## Introduction

**FIGURE 1:**
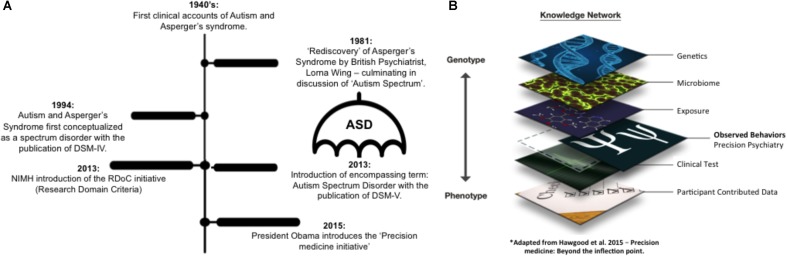
**(A)** While the clinical landscape of Autism has witnessed a transformation with the amalgamation of Autism, Asperger’s syndrome and PDD-NOS into a single umbrella term of autism spectrum disorder, the new era of precision medicine **(B)** encourages the integration of precise individualized information toward a specific diagnosis and intervention https://figshare.com/s/eb6150e5f2fc0acd7a2c.

Autism spectrum disorders (ASD) is an umbrella term encompassing the diagnoses of autism, Asperger’s syndrome (AS) and pervasive developmental disorder – not otherwise specified (PDD-NOS) (American Psychiatric Association, 1994; American Psychiatric Association, 2013). Proposed in the early 1980s (Wing, 1981, 1997; Nordin and Gillberg, 1996), the ‘Autism Spectrum’ was first clinically conceptualized with the publication of the fourth edition of the Diagnostic and Statistical Manual of Mental Health Disorders (DSM-IV, American Psychiatric Association, 1994). This reconceptualization was completed with the publication of the DSM-5, which marked the consolidation of previous diagnostic terminology (American Psychiatric Association, 2013; see **Figure 1A**). However, the amalgamation of formerly heterogeneous disorders under a single diagnostic term was, and arguably continues to be, contentious (Singh, 2011). Clinically, this modification and restriction of diagnostic criteria resulted in an expansion in nosology and epidemiology, raising questions over the latent role of modified diagnostic thresholds (Fombonne, 2003; Hill et al., 2015). In addition, this narrowing of diagnostic terminology, to encompass a broader population under a single diagnostic term, sits in contrast to the current climate of medical advancement, which emphasizes the individualization of diagnosis and treatment. Indeed, the Precision Medicine initiative—including Computational Psychiatry (Friston et al., 2014; Insel, 2014; Wang and Krystal, 2014; Torres et al., 2016)—endorses a personalized and objective approach to health through the integration of layers of the knowledge network to provide a tailored and objective examination of disorder progression, as well as treatment and prognosis, where appropriate (**Figure 1B**). Similarly, the NIMH’s RDoC initiative (Research Domain Criteria; Insel, 2014) encourages research to move beyond broad psychological classifications, such as those provided by the American Psychiatric Association, 2013), which often lack sensitivity and specificity, and instead, traverse populations based on quantifiable physiological factors.

Despite traditionally conceptualized as a behaviorally defined psychological disorder—as mirrored by the lack of biomarkers for ASD—trends reported in 2007 indicated a promising shift in ASD media coverage and academic discourse toward the examination of physiological axes, including underlying neurological etiology (Singh et al., 2007). Such results were further supported by a broad quantification of Autism research focus between 1980 and 2010 by the Interagency Autism Coordinating Center (Office of Autism Research Coordination (OARC) and and National Institute of Mental Health and Thomson Reuters, Inc., 2012). This comprehensive report profiled a 12-fold increase in publication rates, with a hand coding methodology (designed *a priori* to target the dimensions of interest), highlighting the now prominent role of biological and physiological processes in Autism focused research (Office of Autism Research Coordination (OARC) and and National Institute of Mental Health and Thomson Reuters, Inc., 2012). Yet, while inferring a promising shift in the scope of Autism research, the reliance on hand coding and counting techniques employed in both reviews limit the applicability of such results. Specifically, the *a priori* application of user-imposed heuristics to delineate expected thematic clusters, coupled with the subjectivity of hand assessment, artificially constrains and shapes results, rather than allowing self-emerging patterns to be identified.

This article seeks to empirically explore and examine whether broad trends toward a biological and physiological research focus of ASD have continued in light of the consolidation of disorders under the ASD umbrella and recent health initiatives, when no *a priori* expectations or limitations on thematic trends are imposed. To this end, modern bibliometric techniques were empirically informed and coupled with computational metrics, self-clustering network analyses and graph theory visualization methods, to provide accessible summative data of the dynamic evolution and focus of Autism research between 1994 and 2015. Research focus and trends were assessed at two levels; (1) examination of broad trends in research proliferation, journal focus, and citation analytics through objective time-series analytics, (2) comprehensive empirical co-keyword analysis to delineate self-emerging areas of research convergence via modularity statistics.

Acting as an article ‘tag,’ keywords provide a unique and condensed method to visualize areas of research focus (Okubo, 1997), and facilitate substantial data processing. This is in contrast to traditional full-text co-word processing, which often relies on the refinement of concepts via the hand selection of prominent papers or terminology (Callon et al., 1991). Introduced by Callon et al. (1991), and refined by Coulter et al. (1996), these traditional methods of co-word analysis employ a range of methods to sequentially delineate semantic concepts, which are presented and tracked across a 2D space, such as a strategic diagram. However, as outlined above, the use of pre-specified heuristics to isolate internal network parameters raises questions over the objectiveness of such an approach. While underlying association indices are utilized to encapsulate thematic frequency, user manipulation is a feature often adopted in modern bibliometric analysis—from manual semantic coding of core terms to drive subsequent clustering and group identification (e.g., Coulter et al., 1996; Topalli and Ivanaj, 2016; Williams et al., 2016; Isenberg et al., 2017), to the manual manipulation and reduction of large-scale data (Chuang et al., 2013), through to the use of arbitrary thresholds for algorithmic calibration to specify term and group selection (Callon et al., 1991; Coulter et al., 1996; Zhang et al., 2015; Williams et al., 2016). Enforcing *a priori* user-specified heuristics for core elements, such as the number of concepts or keywords that constitute a theme and the number of themes to be identified, artificially constrains, and shapes subsequent clustering. This article departs from this approach, and instead, objectively profiles the evolution and diversity of Autism research with a focus on self-emerging patterns of term association (rather than *a priori* enforced heuristics). Modularity techniques and graph theory visualization provide a comprehensive, accessible overview of self-identified trends in research focus. Network modularity—indicative of research convergence—was self-identified via keyword co-occurrence (i.e., the frequency of keyword co-occurring in research articles across the corpus). However, to enable discussion within the precision medicine context, trends were *a posteriori* contextualized using the broad thematic categorizations of Psychological (author reference to traditional psychological metrics subsuming clinical Psychiatry), Physiological (author reference to biological, or quantified physiological based psychological metrics) or Interdisciplinary. This application of statistics before heuristics marks a departure from traditional hand coding and text-processing techniques. Underpinned by empirical, self-emergent trends as identified across the broad Autism corpus, these emerging patterns enable discussion as to the past, current and future direction of Autism research, within a broader context of new health initiatives.

## Materials and Methods

### Data Collection

A systematic literature search was completed using the core collection of ‘Web of Science.’ Literature with the Topic Field, ‘Autism’ (to reflect pre-2013 term consolidation) was identified and refined based on the following criteria: published between 1994 and 2015 (the year of DSM-IV publication to time of data collection), classified as an Original Research article, published in English and within the United States of America only. This search resulted in a corpus of 17,620 original research articles^1^.

### Data Pre-processing, Keyword Extraction, and Frequency Analysis

Bibliometric information was extracted for each article, including author(s), affiliation(s), publication title, journal, attributed keywords and cited references. This information was formatted and processed within R software (R Core Team, 2016) using functionality within the ‘tm’ and ‘Bibliometric and Co-citation Analysis’ packages (Aria and Cuccurullo, 2017). An initial frequency-based time-series assessment was applied to identify the publication rate per year, the most frequently occurring journals for publication per year, and summative citation analytics. Unfortunately, the provision of author keywords is notably inconsistent—with many failing to provide such text markers—as such, database attributed keywords were extracted to isolate areas of research focus. A total of 25,782 extracted keywords were pre-processed to trim white space and remove numeric only entries before being converted to lower case and stemmed to characters 1:6 (facilitating the automatic augmentation of similar terms, e.g., Behavior, Behavioral, and Behavioral Intervention were subsumed into a single representative term). Duplicates were combined creating a *unique* dictionary of 6242 stemmed keywords for corpus analysis. Tailor-made author software (MATLAB 2017a) generated matrices of database keyword occurrence (frequency) for this unique corpus dictionary, while preserving underlying indexing. A total of 4482 keywords were subsequently removed from the unique corpus due to low occurrence (occurrences less than the median frequency of the database: <6). This refinement resulted in a profile of 1760 unique stemmed keywords (ranging from 7 to 3847 occurrences), which were used for the analytics outlined below. The maximal and minimally occurring keywords across the corpus were also isolated—defined as the 5^th^ and 95^th^ percentile—across the trimmed 1760 keyword frequency distribution, and empirically tracked across the corpus to demonstrate the evolution of research topics (see section “Maximal and Minimal Node Tracking” below and **Supplementary Section 1** for more information and a full list of maximally occurring keywords).

### Levels of Analysis and Assessment

Data was analyzed across the entire corpus, before being split into smaller ‘Decade’ collections to examine any shift in publication focus (Decade 1: 1994–2004 inclusive and Decade 2: 2005–2015 inclusive). In addition, key years were isolated for individual profiling, namely; 1994 as the start year aligned with the publication of DSM-IV, 2015 as the most recent completed year of research, and 2006 which *self-emerged* as a critical landmark signaling the field was poised for transformative change – see section “Trend Analysis: Growth of the Research Field and Thematic Focus” below). Results for the ‘Full Corpus’ and both ‘Decades’ are presented below, while additional analysis of each landmark year can be found in the **Supplementary Material**.

### Examination of Broad Trends: Research Proliferation, Journal Focus, and Citation Analytics

**FIGURE 2:**
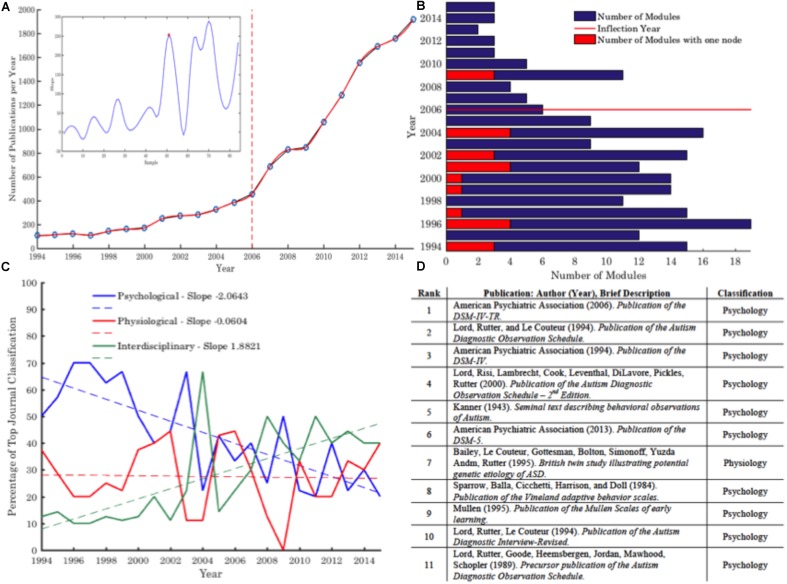
**(A)** Trend analysis of the number of publications under the topic ‘Autism’ per year, and the critical inflection year highlighted (Slope = Δy/Δx; see inset a). **(B)** Overview of the number of self-clustered modules identified across the corpus (see section “Keyword Co-occurrence and Modularity: Research Themes – Evolution and Cross Talk”). A number of yearly modules include a single keyword—these are indicated in red. **(C)** Most frequently occurring journals for publication per year as categorized into broad thematic silos: Psychological, Physiological and Interdisciplinary, and related empirically estimated trend lines. **(D)** Classification of top cited publications across the corpus 1994–2015 demonstrates the prevalence of Psychological research and tools. See **Supplementary Tables 1**–**3** for further information on this classification trend as decomposed to the decade level. For larger high resolution figures please see https://figshare.com/s/5210439deb5a6ba0bcf2.

Research proliferation was initially profiled according to the number of articles identified using the outlined criteria per year. To enable an empirical assessment of the year of inflection (i.e., the critical year whereby research trends demonstrate the largest growth), this data was examined as a time-series trajectory. Specifically, the overarching trend line was subject to cubic spline interpolation—a form of polynomial interpolation—to achieve higher levels of accuracy, from which the derivative of each year/number of publications was extracted, and used to calculate the slope of the trend line of publications (as defined as: Slope = Δy/Δx; see **Figure 2A** inset). The local maxima and minima across the resulting slope trajectory were further extracted, and the first maximal positive difference between local maxima and minima was identified—i.e., the first moment of maximal increase in the slope of the line reflective of an increase in publication rate.

To further contextualize trends of research proliferation in light of the precision medicine platform, the journal of publication was considered, with broad categorization according to three disciplinary silos: Psychological, Physiological focus, or Interdisciplinary. This categorization was performed per year of analysis, with the 100 most frequently occurring journals per year extracted (i.e., those that publish the highest number of ASD research papers).^2^ To further refine this assessment, the top 100 journals were assessed using K-mean clustering (see **Supplementary Figure 1** for depiction). This machine learning clustering algorithm employed minimal squared Euclidean distance to identify groups of closely related observations by randomly inserting placeholders into the data set (user specified *N*), and identifying neighboring clusters. While user enforced *N* specification is required for K-means clustering, this was applied only to identify the most frequently occurring journals, and was empirically justified via systematic exploration of the data settings. For example, if *N* = 2, *N* = 3, and *N* = 4 all resulted in similar group profiles, *N* = 3 was adopted. Thus, an *N* was applied when all neighboring values resulted in similar group profile for the isolation of the demarcated ‘tail,’ representative of the most frequent publications.

The most frequent publications were subsequently classified according to the Journals’ *official description*—extracted from the relevant online presence, and crosschecked (where possible) with Thomson Reuters Journal classification. Terms of focus such as Genetic, Immunological, and Neurobiological were deemed as Physiological, while those such as Education, Intervention, and Mental Health were deemed as Psychological or Clinical Psychiatric in nature. Journal descriptions that explicitly stated interdisciplinary research, or referred to terminology associated with both Physiological and Psychological terms were deemed Interdisciplinary. Authors crosschecked all classification results. While this method involves heuristics, it is applied to enable discussion of research scope and does not impact subsequent modularity techniques employed for keyword analysis. To control for variability in the number of frequently occurring journals per year, the percentage of categorization was calculated. A line of best fit was then empirically applied to the time-series of each categorization distribution, with the slope calculated to allow further trend interpretation and identification of *self-emerging* trends (see section “Trend Analysis: Growth of the Research Field and Thematic Focus” for results).

The references that researchers cite provide a foundation for further discussion or support, and are therefore indicative of research focus or procedures. As such, the above process was repeated for the most frequently cited articles (extracted via K-means assessment of article citation analytics) across the corpus. To enable a broader discussion in light of the precision medicine platform, these were again broadly classified as Psychological, Physiological, or Interdisciplinary in nature. Note, the full reference for each of the top cited articles is provided for reader clarity.

### Keyword Co-occurrence Analytics and Terminology

Acting as an accessible summary of research focus, article keywords were profiled to provide a quantifiable overview of research trajectory, with keyword co-occurrence within a single publication providing insight into broad research themes and convergence. Keyword frequency and indexing were therefore utilized to create weighted keyword co-occurrence matrices across all levels of analysis (Full Corpus, Decades, Yearly) through tailor-made author software (MATLAB 2017a)—with each non-zero entry indicating co-occurrence of *keyword i* alongside *keyword j* within a single publication. The matrix entry (weight) is thus the number of instances keywords occurred together within a single publication across the corpus or years of analysis.

**FIGURE 3:**
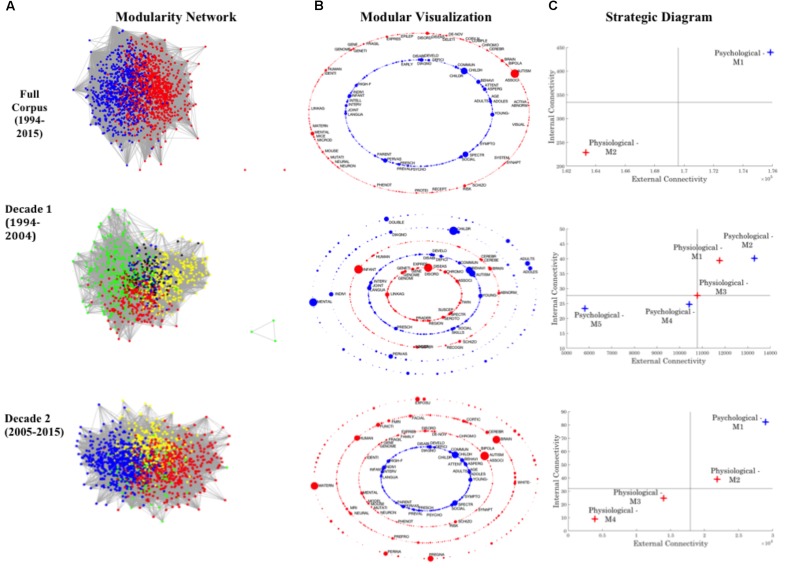
Summary of co-keyword analysis completed across the Full Corpus, and both Decades. **(A)** Keyword co-occurrence matrices are visualized using graph theory methods, and modularity analytics applied to identify *self-clustering* modules. These modules are initially visualized within the context of the overarching network, with each highlighted by a block color (e.g., two modules are initially identified at the full decade, while four are identified for Decade 2). **(B)** To aid accessibility, these modules are visualized as colored concentric rings, each reflecting a module’s singular rank and theme (**Psychological: Blue; Physiological: Red; Green: Interdisciplinary**). Node size reflects normalized Eigenvector centrality – with larger nodes having higher levels of normalized Eigenvector centrality, and thus, empirically assessed as dominant across the module; such prominent internal nodes are labeled (see **Supplementary Section 1** for full node listing used for thematic coding). **(C)** Modular internal and external connectivity metrics were extracted, normalized, and visualized relative to the normalized corpus median values via a strategic diagram. Providing a summary of internal connectivity along the Y-axis, this provides a measure of the cohesiveness of a thematic trend – with highly developed and interconnected themes displaying higher levels of internal connectivity. Summative metrics on the X-axis provide an overview of external connectivity, demonstrating the central dominance of a theme to the research domain. The range of corpus internal and external connections display significant growth between Decade 1 and Decade 2, in line with research proliferation (see **Figure 2**), coupled with simultaneous constriction in modularity, perhaps indicative of the development of cohesive research themes (**Figure 2D**)—see **Supplementary Table 4**. For yearly (1994, 2006, 2015) assessment see **Supplementary Figure 3**. Further, for larger high resolution figures please see https://figshare.com/s/9a0f7b0839fef2df1a6e.

Each unique keyword was visualized within associated network graphs as ‘nodes,’ with keyword co-occurrences represented by a network ‘edge,’ weighted by co-occurrence frequency (see **Figure 3A**). Graph theory connectivity analytics were subsequently utilized to examine the resulting keyword networks and facilitate identification of *self-emerging* corpus modules (i.e., broad research convergence—see **Figure 3A** for color coded modules). Specifically, machine learning modularity analytics drawn from computational neural networks (see Newman, 2006; Reichardt and Bornholdt, 2006) were applied to the weighted co-occurrence matrices to enable the self-identification of network modules. Importantly, while this algorithm may result in slight variability of modularity assignment upon successive trials of implementation, the overarching stability of the broad modular clusters was ensured by repetitive iterations. A network module was defined as a group of nodes (keywords) that are maximally internally connected (co-occurrence between module keywords), and minimally externally connected (minimal co-occurrence with keywords in external modules)—i.e., a *self-emerging* cluster of interconnected keywords. Importantly, in a departure from traditional methods, the number of modules to be identified, and the number of keywords that can constitute a module were not pre-imposed *a priori.*

These self-emerging keyword modules were then ranked to facilitate later visualization using Eq. 1.

Equation 1: Module Ranking

$$R_{I}=N\cdot \left(\overset{N}{\underset{j=1}{\Sigma }}e_{j}\right)$$

Module ranking (*R*_I_) is equal to the product of the number of keywords (*N*) identified within the module times the sum of keyword occurrences (e.g., e_1_…e_M_) across the module. Where, *I* denotes the module ranking index, and index *j* runs through the keywords included within the module, and index *j* runs across the size *N* of the module. Thus, modules with high levels of modular interconnectivity, spanning a large range of keywords, are prioritized.

### Module Thematic Coding: Evolution of the Corpus

Self-clustered modules of co-occurring keywords were thematically coded to visualize the evolution of the research corpus and enable discussion within the broader landscape of precision medicine. To empirically guide this process, the internal importance of each keyword within each module was assessed via eigenvector centrality, as defined by Bonacich (1987). An extension of degree centrality drawn from graph theory, this metric provided a measure of keyword importance according to both the number of connections it establishes to others within the module (i.e., modular keyword co-occurrence), and the central importance of those to which it connects^3^. As such, Eigenvector centrality provides a proportional metric that encompasses the relative sum of a keyword’s co-occurrence neighbor(s) within a module (Bonacich, 1987). The resulting Eigenvector centrality distribution for each module was composed, and the central keywords isolated to guide thematic coding. Central keywords were defined as those within the 95^th^ percentile of Eigenvector centrality as profiled across the module, with this threshold empirically identified as a result of the skewed nature of the underlying distribution of connectivity and keyword frequency. As with the methods outlined above, the origins of isolated central keywords were utilized for thematic guidance, with all coding crosschecked between authors. For consistency, and to aid discussion relative to the current health initiatives, this broad categorization was restricted to: Psychological (including Clinical Psychiatry), Physiological, or Interdisciplinary. While a broad level categorization, the full list of central keywords extracted for each module, and the subsequent thematic coding, is available for the reader in **Supplementary Section 2**.

### Network Module Visualization for Accessible Presentation

To maximize visualization of the data, modules were graphically represented in a concentric ring configuration, with central rings reflecting higher ‘ranking modules’ (as derived via Eq. 1), and each keyword represented as a network node. Modular thematic assignment (Psychological, Physiological, or Interdisciplinary) was coded through node color, while node size was indicative of the internal module importance (as measured via Eigenvector Centrality). To enable comparison across all modules at the level of analysis (i.e., Full Corpus, Decades, Yearly), all values of Eigenvector Centrality were normalized via Eq. 2.

Equation 2: Normalization of Parameters

$$P_{n}=\frac{P_{o}-P_{\min}}{\left(P_{\max}-P_{\min}\right)}$$

*P*_n_ represents the normalized parameter value, *Po* is the original parameter value, and *P*_max_ and *P*_min_ are the maximum and minimum parameter values derived from each level of analysis.

### External and Internal Connectivity Across Modules – Relative Importance of Network Modules

The *External* and *Internal Connectivity* of self-clustering module keywords were calculated according to Eqs 3 and 4, respectively. The modular external connectivity provided a summative metric of the total *external links* associated with a module. This provides a summary of the links present between keywords within the module (e.g., *Keyword i*) and external keywords in neighboring modules (e.g., *Keyword h*), with higher levels of module external connectivity indicating a high number of external connections established to neighboring modules.

Equation 3: Modular External Connectivity

$$EC=\overset{N}{\underset{j=1}{\Sigma }}e_{ih}$$

In contrast, the internal connectivity metric provides a summary of the number of internal links within a module, such as the number of links between *Keyword i* and *Keyword j* as denoted by *e_ij_*, where the number of keywords within the module is denoted by *N*.

Equation 4: Modular Internal Connectivity

$$IC=\frac{\overset{N}{\underset{i,j=1}{\Sigma }}e_{ij}}{N}$$

Modular internal and external connectivity co-occurrence metrics were normalized across the level of examination according to Eq. 2, and visualized to consider the role and prominence of modules across the corpus. Specifically, these normalized connectivity metrics for each module were plotted on a strategic diagram (Callon et al., 1991; Coulter et al., 1996), and viewed in relation to the median values of module internal and external connectivity across the network corpus. Self-clustering thematic modules with higher levels of internal connectivity across the corpus are highly interconnected and well-established. In contrast, those with higher levels of external connectivity provide keywords that are areas of research ‘cross talk,’ connecting to neighboring self-clustering modules.

### Areas of Thematic ‘Cross Talk’: Co-occurrence Between Modules

As outlined, levels of external connectivity were profiled for each keyword across the self-emerging modules – with this metric reflective of the number of connections that a keyword establishes with those in neighboring modules. These external connections can be formed with neighboring modules of either the same (intra-thematic) or alternative (inter-thematic) thematic coding. Connections forming across themes (inter-thematic) act as junctures or points of ‘cross talk’ that tie domains together. To this end, levels of external connectivity were normalized (via Eq. 2) across each level of analysis to provide a normalized external metric (NEM). Module keywords with a normalized external connectivity level above the 95^th^ percentile of NEM were isolated, and associated co-keyword frequency metrics extracted, and systematically ranked. Associated partner keywords (i.e., the external keywords to which these internal module keywords make connections) were subsequently identified. From this, an isolated co-occurrence matrix was created and visualized via a color-coded matrix, allowing the identification of core points of modular and thus thematic ‘cross talk.’

### Probing Network Stability

The resilience and stability of the corpus and self-clustering modules was examined via removal of *network hubs*. Network hubs were defined as maximally occurring keywords across a corpus. Such terms may mask nuanced connections with less prominent keywords across or within thematic modules, and as such were removed to probe the stability of the self-emerging networks. To this end, keyword frequency was profiled across levels of the corpus, with those keywords constituting the top demarcated group (as empirically identified via k-means (*N* = 3) see **Supplementary Figure 1**) removed from the corpus. Again, this user specified value of *N* = 3 was identified upon systematic exploration of settings—*N* = 2, *N* = 3, *N* = 4 resulted in similar group profiles at the full corpus level, therefore *N* = 3 was adopted for consistency across all levels.

Through this method and across all levels of corpus analysis (Full Corpus, Decades), the following keywords were empirically identified using k-means clustering as network hubs (maximally occurring) and thus removed from the corpus: Asperger, Autism/Autistic, Child (and all variations such as Children, Childhood), and Spectrum. Self-clustering modularity metrics were again processed and visualized, as derived from this refined corpus, allowing consideration of the stability of internal structures and thematic re-organization or prominence.

### Maximal and Minimal Node Tracking

Maximal (83 keywords; see section “Data Pre-processing, Keyword Extraction, and Frequency Analysis” above) and minimal nodes were tracked across the research corpus to consider changes in prevalence via time-series analytics (see **Supplementary Section 1** for full list). This more in-depth consideration of research focus was refined to systematically profile, and individually track, a core group of 12-prominent (frequency based) keywords. This individual tracking was completed after removal of network hubs (as outlined in Section “Probing Network Stability”), and the amalgamation of related or interchangeable terms to minimize redundancy.

## Results and Discussion

### Trend Analysis: Growth of the Research Field and Thematic Focus

A total of 17,620 articles were isolated through the systematic search outlined above, of which 13,513 had database-attributed keywords used for thematic trend analysis. Examination of the number of publications per year (**Figure 2A**), illustrates a strong trend of research proliferation—with an average publication growth rate of 14.74%. This trend analysis was further quantified to isolate the year of research expansion—a ‘critical point’ in the evolution of Autism research. Defined as the first point of maximal difference between local minima and maxima (i.e., the first point of maximal *increase* in calculated slope representing underlying change in publication rate), 2006 was identified as the inflection year (**Figure 2A** inset). However, despite this general increase in Autism publications across the timeframe, self-clustering analytics demonstrate a simultaneous reduction in the number of modules self-emerging per year (**Figure 2B**), indicative of a convergence of research discourse.

Classification of journal focus by broad disciplinary silos (Psychological, Physiological, Interdisciplinary) demonstrates a potential underlying shift in research focus. A clear downward trend in research published within Psychological journals can be identified, along with a growing prominence of research published within Interdisciplinary focused journals. Interestingly, despite being characterized as a psychological construct, the trend for Physiological research (as inferred via journal focus) has remained relatively consistent across the timeframe (**Figure 2C**). However, despite the downward trend in publications within psychological journals, results indicate that the most frequently cited articles across the research corpus retain a psychological stance (**Figure 2D**). This would imply that psychological terminology and constructs continue to play a role in emerging Interdisciplinary and Physiological research—a trend that continues across both decades of analysis, see **Supplementary Tables 1**–**3**. Thus, despite promising trends for a shift toward physiological axes of Autism, potentially reflecting neuroscientific and genetic efforts to delineate quantifiable biological markers, psychological and psychiatric terms, and related methodologies, appear to dominate and influence the foundations of research across the corpus.

### Keyword Co-occurrence and Modularity: Research Themes – Evolution and Cross Talk

Modularity analytics were applied to the weighted co-occurrence matrices extracted for keywords across each level of the corpus (Full Corpus, Decades, Yearly). The resulting self-clustered modules were ranked according to Eq. 1, and the underlying connectivity metrics of each were extracted for thematic coding. The below provides a brief overview of this analysis for the complete networks.

#### Full Corpus (1994–2015 Inclusive)

Examination of the Full Corpus (1994–2015) identified two *self-emerging* clustering networks through modularity analytics, which were broadly categorized into two discrete thematic silos: Psychological and Physiological (**Figures 3A,B** – see **Supplementary Section 2** for isolated keywords used for thematic coding). Despite being comprised of fewer individual nodes (see **Supplementary Table 5** for network characteristics at all levels), the Psychological module displays prominence across the corpus, with notably higher levels of internal and external connectivity relative to both the corpus normalized median, and the associated metrics of the neighboring Physiological module (**Figure 3C**). This would imply a well-established field of Psychological research that acts as a ‘driving’ force within the broader context of Autism discourse. While this trend persists across the Decade level of analysis, results also demonstrate the growing momentum of Physiological research in Decade 2, with an increase in sub-themes.

**FIGURE 4:**
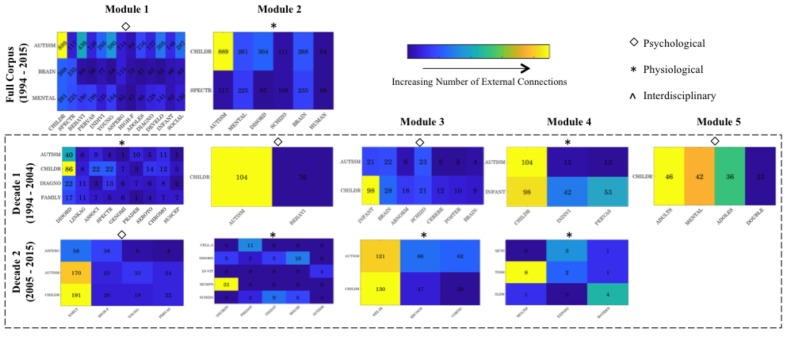
Summary matrices visualizing the external connections between modules identified across each level of the corpus. Prominent internal module keywords are indicated on the x-axis, while primary external module partner keywords are represented on the y-axis. Note external partner keyword (y-axis) can appear more than once across multiple external connections for individual modules. For instance, the term Brain acts as a prominent external connection for more than one module, and thus is a core network hub enabling multiple module connections. Matrix colors are indicative of normalized levels of external connectivity, with lighter colors denoting a larger number of normalized connections (original count data is also indicated). The top panel provides an overview of the Full Corpus modularity analytics demonstrating cross talk between the psychologically themed M1 and physiologically themed M2. The two lower panels illustrate Decade 1 and Decade 2 modularity analytics respectively, demonstrating external connections between core modules. While Decade 1’s self-identified modules appear to display nuanced levels of integration in M1 and M3, closer inspection demonstrates these are limited to arguably higher-level external descriptor terminology such as ‘Autism’ and ‘Children.’ Further, Decade 2 M2 and M4 display a range of external junctures, yet these appear to be largely intra-thematic. For yearly (1994, 2006, 2015) assessment see **Supplementary Figure 4**. For larger high resolution figures please see https://figshare.com/s/3edd5690135926764d04.

Specifically, Decade 1 analytics reveal the clustering of modules deemed Physiological according to research focus on Genetic/Genomic, and Cerebellar/Neurological factors, which are expanded further in Decade 2 via the inclusion of Maternal or Prenatal factors associated with Autism. However, despite this increase in Physiological thematic focus, modular interconnectivity continues to demonstrate the prominence of Psychological modules across both Decades (**Figure 3C**). Further, examination of junctures of thematic cross talk between modules highlights the prominence of high-level descriptor terminology, such as Autism and Children, in creating research interconnectivity across the broader arena (**Figure 4**). As illustrated in the color-matrix of external connectivity, both Psychological and Physiological networks display restricted thematic interconnectivity, with primary junctures largely isolated to such high-level group descriptors located in each module. Initial interpretation may infer inappropriate terminology clustering (i.e., the mistaken inclusion of high-level descriptor terminology within Physiological themed networks). However, high-level descriptor terms identified within Physiologically themed modules are, by virtue of the network algorithm, maximally connected to internal modular Physiological co-keywords. Results thus indicate the role of these network hubs in the consolidation of the broader research arena—providing junctures to create cohesiveness between Psychological and Physiological terminology and research—as well as their role in consolidating each individual research arena.

### Stability of the Network Upon Removal of Core ‘Hubs’

Modularity co-keyword analytics demonstrate the influence of Psychological research, while also indicating a move toward Physiological themes. However, the persistent trend for prominent interconnectivity located in high-level descriptor terminology questions the quality and level of interconnectivity between thematic areas, and the dominance of Psychological clusters. Specifically, the dominance of descriptor terminology may serve to mask underlying nuanced connections between keywords and modules, and thus impact modular self-clustering. Removal of these network hubs resulted in an intuitive reduction of the range of internal and external connectivity (**Supplementary Table 4**), and a notable drop in the corpus median across all levels of analysis—allowing expansion of the module networks.

#### Full Corpus (1994–2015 Inclusive)

**FIGURE 5:**
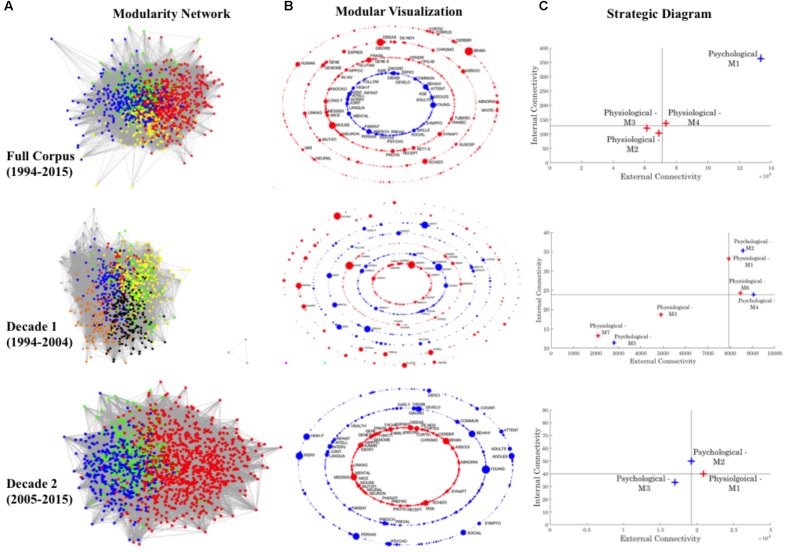
**(A)** Refinement of the network after the removal of core network hubs to assess the stability of network clusters and modularity at all levels of examination. As demonstrated in **(B)**, these are classified into Psychological, or Physiological focus based upon core internal nodes. This categorization is color coded (**Psychological: Blue; Physiological: Red, Green: Interdisciplinary**), with the order of concentric rings reflecting each module’s rank. In addition, this corpus was systematically deconstructed to consider patterns across two individual decades (Decade 1: 1994–2004 and Decade 2: 2005–2015), and internal and external connectivity metrics **(C)**. Intuitively, the removal of network hubs leads to reduced levels of overarching median normalized external and internal connectivity at all levels. See **Supplementary Table 4** for comparisons. For larger high resolution figures please see https://figshare.com/s/7f723b2c983a747f7e44.

Removal of network hubs resulted in an expansion of modules across the Full Corpus from a discrete Psychological and Physiological thematic cluster to four distinct modules (**Figures 3B**, **5B**). In particular, the removal of core network hubs—all of which were deemed as high-level descriptive terminology—resulted in the dissolution of the single Physiological module, into three distinct networks all with a Physiological thematic trend (**Figure 5B**): M2 Genetics and Mouse Models, M3 Genetics and DNA, and M4 Neuroscience or Neurological Factors. Levels of internal and external connectivity across these refined modules demonstrate the continued dominance of the Psychological module in terms of both internal cohesiveness and external connections to the neighboring Physiological modules (**Figure 5C**).

**FIGURE 6:**
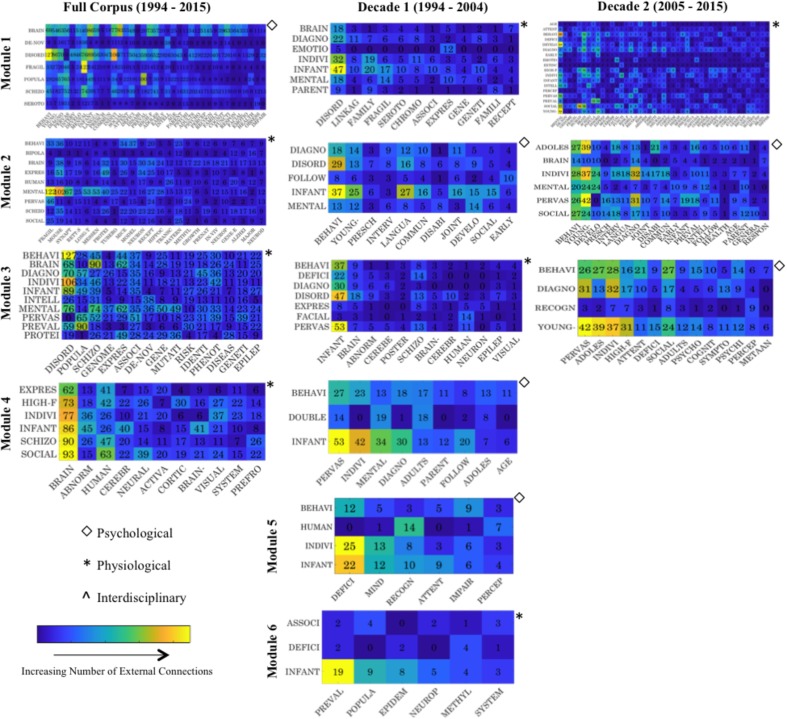
Summary matrices visualizing the external connections between modules within the refined network, i.e., network trimmed of corpus descriptor hubs such as ‘Autism.’ Prominent internal module nodes (keywords) are indicated on the x-axis, while core external partner nodes are represented on the y-axis. Colors are indicative of normalized levels of external connectivity, with lighter colors denoting a larger number of normalized connections (note: original count data is indicated. The left panel provides module networks for the Full corpus, while the two right panels provide those for Decade 1 and Decade 2 respectively). For larger high resolution figures please see https://figshare.com/s/aa56fdd878db4a04a521.

When examined considering levels of modularity cross talk, the Psychological module appears to make a range of connections with external Physiological term landmarks including: *de novo*, Fragile X, and Neurotransmitters (Serotonin). However, the somewhat descriptive term, ‘Brain,’ is identified as the main area of thematic overlap driving connectivity factors between modules (**Figure 6**). While Physiological modules (M2–M4) display lower levels of overall external connectivity (**Figure 5C**), prominent nuanced junctures are created (**Figure 6**). Interestingly, however, the more ‘restricted’ module (M4) demonstrates the highest levels of connectivity (**Figure 5C**) in relation to the more nuanced M2—perhaps a by-product of the dominance of the descriptor Psychological terms to which it forms junctures (**Figure 6**)—again illustrating the importance of such Psychological research themes. In sum, removal of core network hubs results in notable fragmentation in research thematic cohesiveness, which specifically targets the Physiological strand of academic research.

#### Decade Examination

Removal of core hubs continues to impact self-clustering modularity during both Decade 1 (1994–2004) and Decade 2 (1995–2005).

##### Decade 1

Similar to results at the Full Corpus level, the removal of descriptor terminology in Decade 1 fragments the Physiological thematic network (**Figure 3B**)—increasing the number of modules from two to four (**Figures 3B**, **5B**). Sub-group thematic trends demonstrate specific Physiological sub-modules related to Genetic (M1) and Neurological (M3) research—mirroring those present at the complete (hub terminology included) Decade 1 level—with two additional thematic clusters now identified focused on Pharmaceutical topics: MMR Vaccination (Measles, Mumps and Rubella – M6) and the administration of Risperdal (M7). Viewing these sub-cluster thematic trends within the context of corpus analytics further demonstrate this evolution. Specifically, while Psychological constructs remain largely dominant, Physiological modules M1 and M6 display high levels of internal and external connectivity (**Figure 5C**) during Decade 1, inferring dominance in the wider research domain across this period. As such, results indicate that network hub descriptor terms play a key role in the cohesiveness of Physiological research clusters during Decade 1, and their removal reveals a nuanced pattern of emerging strength in this area of research.

##### Decade 2

In contrast to the trend of Physiological fragmentation of Decade 1, the removal of network hub descriptor terminology across Decade 2 has a consolidation effect on Physiological modules—now causing fragmentation within the Psychological module (**Figures 3B**, **5B**). As a result, this descriptor terminology is now core to the cohesiveness of the dominant Psychological modules—rather than Physiological modules in contrast to Decade 1. Combined this may imply greater refinement and consolidation of Physiological research, and a parallel dissolution of Psychological research. Indeed, corpus analytics illustrate that the tenuous strength of Physiological modules in Decade 1 has solidified—leading to a new dominance of Physiological research in Decade 2, with higher levels of external cohesiveness driving the broader research arena (**Figure 5C**). In contrast, the growing dissolution and insularity of Psychological research is highlighted via the dominance and impact of higher-level group terminology (network hubs) during Decade 2, and the lower levels of external connectivity indicative of a weakened research area. However, it should be noted that despite the insularity of the Psychological module, and comparable growth in Physiological dominance, lower levels of internal connectivity of the Physiological module infers a need for further consolidation of this research field. This may be reflective of the disparate nature of Physiological constructs and research to date, and may be a contributing factor in the slow emergence of the physiological Autism research domain.

Examination of areas of thematic cross talk upon removal of high-level descriptor terminology also illustrates a more nuanced level of interconnectivity. New junctures or thematic intersections now include terms such as Brain, Fragile, Disorder, Prevalence, Mental, Infant, Diagnosis, Individual, and Behavior (**Figure 6**—which are subsequently profiled in detail in Section “Tracking Top Nodes – Decomposing the Thematic Evolution to Primary Research Areas”). Detailed examination of Psychological modules that retain dominance across each level of analysis (**Figure 5**) further infers a level of intra-thematic insularity inflating metrics, with such clusters subsuming terms such as Behavior, Language and Intervention, yet showing minimal external thematic cross talk (**Figure 6**).

### Tracking Top Nodes – Decomposing the Thematic Evolution to Primary Research Areas

The growing prevalence of Physiological research, as examined via co-keyword coherence metrics, raises questions as to the specific research focus of the field. As such, the maximally occurring nodes from across the corpus (as defined as >95^th^ percentile—see section “Maximal and Minimal Node Tracking”) and related minimal nodes (<5^th^ percentile) were automatically isolated and tracked across the timeline of the corpus (**Supplementary Section 1** for full list and **Supplementary Figure 2** for timeline tracking). A sub-cluster was subsequently identified to represent core *research topics* that are prevalent across the corpus.

**FIGURE 7:**
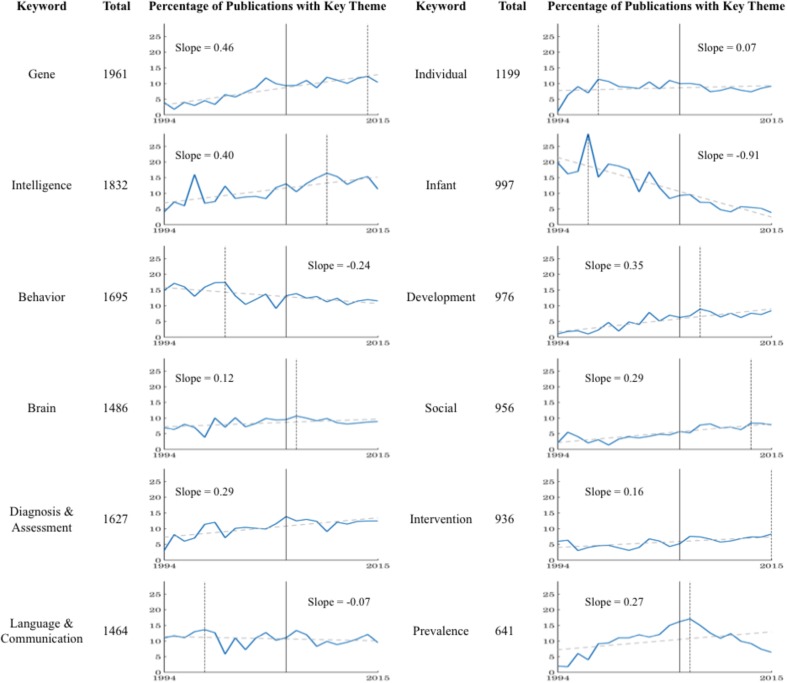
Systematically profiling and tracking of 12 prominent keyword/themes across the corpus. The count of each theme within the maximal nodes per year was isolated, and transformed into a percentage of the total year publications – providing a standardized metric. The inflection year (2006; see **Figure 2**), and the year of maximal occurrence for that theme (as denoted by the dashed vertical line) are referenced for contextual understanding. In addition, a line of best fit is empirically derived and illustrated for each theme, demonstrating the continued growth across the academic arena for all themes with the exception of Infant, Behavior and Language and Communication. For larger high resolution figures please see https://figshare.com/s/a98a22865ef50b9b278b.

These core research topics emerged by trimming the corpus maximal keyword list of high-level descriptor terminology, as outlined above, and then amalgamating associated terminology to minimize redundancy. Specifically, Mental Retardation (now a disputed term) was amalgamated with Intelligence Quotient (IQ) and Intelligence to form an overarching ‘Intelligence’ area. Similarly, Diagnosis was combined with Assessment, Identification and reference to the DSM (all variations) to form ‘Diagnosis and Assessment.’ Finally, Language and Communication were collapsed, as were Intervention and Therapy, and Gene and Genome. A total of 12 prominent keywords were subsequently identified through the application of the K-means clustering algorithm for systematic profiling and tracking, namely: Behavior, Prevalence, Intelligence (originally Mental Retardation), Brain, Individual, Gene, Diagnosis, Infant, Development, Social, Intervention and Therapy, and Language and Communication (**Figure 7**).

The 12 themes display a bias toward Psychological terminology. Specifically, of these terms only Gene and Brain are clearly identified as having a Physiological basis. Of these, substantive growth is noted in the term Gene—subsuming Genetic, Gene, and Genome—perhaps demonstrating a gradual move toward an increase in Physiological and Interdisciplinary research focus. Furthermore, in line with this evolution of research focus, declining constructs include Language and Communication, and Behavior (both of which experienced peak occurrences prior to the inflection year of 2006), with Infant identified as the maximally declining prominent keyword across the corpus. Such trends support a broadening scope of Autism research, with a gradual departure from a strong psychological focus and isolated fields of inquiry, such as Language and Communication, toward a more refined examination of outcome metrics. However, in all instances, it should be noted that despite such decline in prominence, these terms remain within the group of *maximally occurring* themes across the research corpus, and thus retain dominance and strength in the broad research field—again indicative of a continued Psychological framework.

## Conclusion

Traditionally, analytical text review techniques employ a range of heuristics that can limit the ability to systematically quantify the inherent dynamics of research trends. While heuristics are often required to enable meaningful discussion and interpretation of data, the order of application is critical. The *a priori* application of heuristics can result in user-specified criteria that artificially shape results, raising questions of the validity of such an approach. This is often illustrated in user imposed heuristics such as prior manual hand coding to cherry pick thematic modules that are to be delineated across a corpus (Coulter et al., 1996; Office of Autism Research Coordination (OARC) and and National Institute of Mental Health and Thomson Reuters, Inc., 2012; Topalli and Ivanaj, 2016; Williams et al., 2016; Isenberg et al., 2017), pre-specification of the number of modules or themes to identify and pre-setting the number of words that can be used to form a module (Callon et al., 1991; Coulter et al., 1996; Zhang et al., 2015; Williams et al., 2016). These methods inevitably impact the utility of high-end mathematical text processing techniques and programs. This review marked a departure from such methods by allowing self-clustering modularity to be identified across the broad research arena *prior* to the inclusion of any user-imposed heuristics to facilitate discussion. Specifically, augmenting text-processing techniques with network and graph theory enabled an empirical profiling of research convergence as denoted by the co-occurrence of keywords. These broad research modules were further contextualized by *a posteriori* broad categorization relative to new health initiatives. Thus, user-imposed criterions were either empirically justified through the systematic exploration of settings (e.g., in the setting of *N* thresholds for frequency distributions), or were applied *a posteriori*, driven by empirical self-emerging clusters. This use of empirically informed, data-driven methods to define user heuristics ensured minimal subjectivity.

This self-evolving heuristic free principle of modularity analytics to identify research focus, coupled with the empirical examination of publication trends using time-series analytics, demonstrated a trend of research proliferation—in line with increased public awareness and incidence rates of Autism (Fombonne, 2003; Matson and Kozlowski, 2011)—and resulted in the identification of core landmarks demarcating prominent areas of interest across the field. These automatically identified results include: (1) 2006 as the year of inflection for research proliferation, (2) the continued role of Psychological and Psychiatric terminology, and thus methods, across the broader Autism research arena, and (3) the automatic identification of underlying modules reflective of historical contention and debates within the general Autism community, such as the MMR scandal and the authorization of Risperdal for the management of behavioral difficulties.

### Autism Research Over the Years: Growth and Broad Trend Analytics

Empirical examination of the rate of publications across the broad Autism field facilitated automatic identification of 2006 as the year of critical change—the inflection point—where research demonstrated an accelerated rate of change. While in line with previous results inferring the importance of 2005–2006 as derived via parsimonious methods, such as hand counting and subjective interpretation (see Office of Autism Research Coordination (OARC) and and National Institute of Mental Health and Thomson Reuters, Inc., 2012), this quantifiable, mathematical examination of rates of publication provides a robust methodology, with an automatic landmark isolated. Contextualization of this self-identified landmark highlights the potential role of the Combating Autism Act passed in 2006 by then President George. W. Bush. This Act facilitated the provision of funds via the NIH to support research efforts that sought to ‘*develop and implement a strategic plan to conduct and support autism spectrum disorder research*’ (full Act available here: https://www.congress.gov/bill/109th-congress/senate-bill/843). With an increase in United States funding, it is perhaps unsurprising that 2006 marked the beginning of increased research interest within the American research community. Furthermore, this four-step program explicitly included promoting research into the development and validation of reliable screening tools—perhaps a driving factor in the growth and persistence of core research themes, such as Diagnosis and Assessment, Prevalence and Intervention—as illustrated in the individualized examination and tracking of core research topics.

Broad trend analytics further illustrate a general trend for a decline in publications within journals of a predominantly Psychological focus, and a parallel growth in publications within journals with a Physiological and Interdisciplinary focus. Conversely, the empirical assessment of prominent citation analytics across the corpus infers a continued dominance of psychological constructs and tools. Specifically, these analytics suggest a broad research grounding upon Psychological constructs and knowledge, not limited to the seminal works of Kanner (1943) and Asperger (1944). Rather, the dominant cited work across the corpus are that of observational psychological or psychiatric tools often used to quantify behaviors for diagnostic and symptomatology characterization purposes, e.g., ADOS (Lord et al., 2000), ADOS-2 (Lord et al., 2012), Vineland scales (Sparrow et al., 1984), and variations of the DSM (e.g., American Psychiatric Association, 1994, 2009, 2013; American Psychiatric Association, 2013). The prevalence of citations to such observational based tools across the corpus infers their central role within the broader Autism research domain. Yet, despite the recent modifications in the clinical conceptualization of Autism serving to magnify the heterogeneity of individuals on the spectrum, the prevalent diagnostic methods and tools remain arguably stagnated (see **Figure 1**), questioning the ability for these observational, behavioral based tools to be integrated into the broader context of computational psychiatry and precision medicine. So what role do such clinical tools play in the broader research arena? With a characteristic symptomatology profile and diagnostic procedure strongly—and historically—entrenched within the Psychological field, this broad application may imply the field of academic Autism enquiry draws on such metrics and constructs to inform and guide underlying research questions, outcomes and methodologies. Further, the continued prevalence of Psychological constructs and tools, despite a general trend toward growing Interdisciplinary and Physiological research, poses the possibility that Autism research continues to adopt these dominant behavioral paradigms to provide behavioral markers on which to map physiological correlates and substrates. Indeed, psychological and psychiatric metrics such as the DSM and ADOS are often the first step in Autism clinical research—arguably of any nature—providing the pre-defining label criteria to stratify participants by diagnostic terminology. It is this stratification of participants by broad and arbitrary thresholds from DSM criteria, using subjective clinical observational techniques that negate the physiological axes of development (Hofmann, 2014), that subsequent analytics such as neuroscientific, genetic, or indeed, behavioral, must map onto. However, as noted by Thomas Insel, ‘DSM diagnoses are based on a consensus about clusters of clinical symptoms, not any objective laboratory measure,’ thus while adopting modern techniques in conjunction with arguably arbitrary clinical thresholds, we often ‘reject a biomarker because it does not detect a DSM category’ (Insel, 2013). Setting the context for the current RDoC initiative, these criticisms of DSM diagnostic measures, and related psychological and psychiatric tools that seek to operationalize such categorization, highlight the limitations of advancing physiological and biological research using these methods. Thus, despite an initial quantification of journal focus inferring a growth in Interdisciplinary assessment, and an arguably consistent focus from a Physiological stance (**Figure 2C**) in line with previous trend assessments (Singh et al., 2007; Office of Autism Research Coordination (OARC) and and National Institute of Mental Health and Thomson Reuters, Inc., 2012), these broader results point to a potential tension between clinical (Psychological and Psychiatric) and Physiological based basic science approaches to Autism; a tension that is elucidated further through assessment of broad co-keyword thematic trends.

### Connectivity Network Analyses and Machine Learning Methods: Unveiling Thematic Tension

The automatic identification of landmarks and thematic change across the Autism research corpus invites the use of these renovated bibliometric methods to aid problem identification within specific sub-areas of a general theme. For example, this self-emerging analytical model of research convergence further illustrates potential tension between psychological or psychiatric approaches and physiological methods toward the study of Autism. Specifically, thematic coding of self-emerging research modules into broad silos (Psychological, Physiological, or Interdisciplinary) demonstrates the continued dominance of psychological constructs and terminology across the research domain. This is reflected in the internal and external cohesion of these thematic clusters across levels of analysis. However, more nuanced examination relative to the decade evolution reveals a gradual prevalence and consolidation of physiologically focused research—with initially disparate terms of Decade 1 (1994–2004) covering sub-themes such as Genetic, Neurological, Pharmaceutical and Prenatal axes of Autism, converging by Decade 2 (2005–2015).

Further novel metrics facilitated examination of the implicit struggle between Psychological and Physiological defining thematic constructs associated with Autism by consideration of inter- and intra-thematic keyword interconnectivity. These metrics of interconnectivity repeatedly demonstrated the latent role of Psychological terminology in consolidating early research (Decade 1). Specifically, broad group descriptor terminology acts as a cohesive landmark or juncture between early disparate Physiological themes, particularly during Decade 1 (1994–2005). It is not until Decade 2 (2005–2015) that this consolidator role of Psychological terminology in Physiological research is minimized—with the removal of descriptor terminology having a minimal impact on Physiological modularity. Such results are illustrative of the growing cohesiveness of this emerging research arena, with internal and external connectivity metrics now identified at more nuanced levels. By Decade 2 this high-level network hub descriptor terminology provides cohesion to Psychological modules—illustrative of growing insularity (a decrease in inter-thematic trends), dissolution (fragmentation), and reduced dominance as profiled in the corpus strategic diagram.

This implicit struggle in terminology and focus inferred by both self-clustering modularity analytics and supported by broader research categorization and citation analytics, again points to the innate struggle currently evidenced in the Autism clinical and research domain. This evolving physiological thematic dominance may reflect mounting questions raised over the role of broad DSM criteria in light of recent health initiatives, such as the RDoC (Insel, 2009; Insel et al., 2010) and Computational Psychiatry (Insel, 2014). The recent health initiatives attempt to operationalize and resolve tension between clinical observational methods defining Autism, and the emerging body of knowledge that points toward the physiological underpinnings of Autism as quantified by scientific and engineering disciplines. Indeed, federal and state agencies across the United States now encourage the use of objective biometrics, machine learning methods and artificial intelligence, as outlined in the associated funding priorities (Insel et al., 2013); a focus that also extends to private foundations^4^. These new initiatives are paired with a fast-growing wave of open access repositories aimed at examining signals from the nervous systems (e.g., the Autism Brain Image Data Exchange repository, the National Database for Autism Research, The NIMH/NIH Data Archive, among others).

### Autism in the United States vs. the Rest of the World

The present work constrained the search criteria to the United States owing to the fundamental differences that exist in health system policies and legislation between the United States, Europe and the rest of the world. Such differences shape the type of research, public policies and legislations that drive diagnostics and treatment criteria in Autism and related neurodevelopmental conditions. As such, extending the search to the scientific literature derived from research in other countries would have posed the additional challenge of considering different policies across different nations. Our work aimed at first gaining an understanding of the landscape of autism research in the United States, and then begin the process of evaluating how the outcome of this search would compare with the search that would include scientific literature from other nations, i.e., with different policies and diagnostic criteria. In this sense, the results reported here may serve as a baseline to measure departure in research from other countries with different insurance coverage policies and medical systems, relative to the United States case. We reasoned that perhaps taking this quantitative approach to express and characterize the prevalence of subjective observational criteria may alert the United States system of the unmet needs of scientists working on autism and in this way, begin to explore new horizons by considering approaches in other nations.

Outside the United States, taking a more holistic approach, i.e., one that is inclusive of both mental and physical states of the person, is not new. The ICD-10 (World Health Organization, 1994), the core medical listing of ‘disease and related health problems’ published (open access) by the World Health Organization^5^, provides a comprehensive encyclopedia of both psychological and physiological conditions. Updated yearly, the entry for Autism can be found under section F80–F89, under ‘Disorders of Psychological Development’ of the Mental and Behavioral Disorders: Diagnostic Criteria^6^. This block section, covering Autism and additional syndromes of development, is prefaced with the following statement:

‘The disorders included in this block have in common: (a) onset invariably during infancy or childhood; (b) impairment or delay in development of functions that are *strongly related to biological maturation of the central nervous system; … affected include language, visuo-spatial skills, and motor coordination…’* (World Health Organization, 2016; see: http://apps.who.int/classifications/icd10/browse/2016/en#/F80-F89). Emphasis added by Author.

While this preface is in reference to a broader set of developmental disorders than Autism alone, it sits in contrast to the DSM, published and approved by the American Psychiatric Association (1994). Published to operationalize the working symptomatology of mental disorders listed in the ICD-10, this manual omits these Physiological features—in line with a Psychological perspective. Furthermore, with the publication of DSM-5 there has been growing concern expressed over the potential of the DSM criteria of ‘overmedicalizing normal human behavior’ (Watts, 2012), and the existence of latent conflict of interests between APA and pharmaceutical companies that utilize DSM criteria to delineate disorders for drug treatment. Most notably, this profound conflict of interest led to the establishment of the Physician Payments Sunshine Act (2010), which aimed at increasing transparency of financial relationships between health care providers and pharmaceutical manufacturers at large. Within the psychological and psychiatric arena this served to highlight conflicts of interest existing across the American Psychiatric Association (Cosgrove et al., 2006, 2009a,b, 2014a,b; Cosgrove and Krimsky, 2012). The notoriety, and controversy surrounding the DSM, most famously the DSM-5, point toward broader discontentment across the academic arena. In this sense, the emerging trend of interdisciplinary research that our study revealed coincides with a new transformative path toward Computational Psychiatry. This emerging discipline relies more on mathematically driven methods analyzing physical data than on observational techniques largely based on opinion.

### Automatic Identification of Historical Contention and Public Discourse

Akin to trends inferred by Singh et al. (2007) review of Autism public discourse, self-clustering trends isolated across the timeframe—particularly those mirroring Prenatal and Pharmaceutical facets—also point at researchers’ attunement to these public policy, societal issues, and funding agendas surrounding Autism. For instance, a specific Physiologically coded module with prominent nodes relating to Measles, Mumps, Rubella, and Prevalence self-emerges across examination of research during Decade 1 (1994–2004). With high levels of internal and external dominance across the Decade corpus, this module reflects the growing public discussion and alarm, in light of a now discredited theory by Andrew Wakefield in 1998 of a causal link between the MMR vaccination and the onset of Autism. Similarly, a second Physiological module with prominent nodes relating to double-blind studies of Risperdal is self-identified during Decade 1. The U.S. Food and Drug Administration (FDA) authorized Risperdal, the trade name for the antipsychotic drug Risperidone, for the treatment of Schizophrenia symptomatology in 1993 (see current label information, FDA, 2006). Research into the use of this pharmaceutical intervention for Autism was examined in prominent studies such as McCracken et al. (2002), with Johnson and Johnson subsequently filing an FDA petition for the use of Risperdal for the treatment of irritability in Autism—which was approved for use in children, aged 5–16, in 2006 (FDA, 2006). This pharmaceutical intervention, now commonly acknowledged to come with a range of side effects^7^, is currently authorized for use to treat Autism in children, Schizophrenia and manic episodes in those that suffer from Bipolar disorder (FDA, 2007). However, a range of recent lawsuits (U.S. Department of Justice, 2013a,b) indicates the off-label use of this medication for ADHD. When viewed in light of recent changes in clinical diagnostic criteria allowing (for the first time) co-morbidity of Autism and ADHD (DSM-5, American Psychiatric Association, 2013), future questions may be raised as to the implications of pharmaceutical interventions and current diagnostic thresholds. Indeed, recent examination of vast data repositories such as ABIDE (Autism Brain Imaging Data Exchange) points at the pervasive use of psychotropic medications in autism and ADHD, and the potential relationship relative to physiological measurements of motor control (Torres and Denisova, 2016; Torres et al., 2017).

### Autism Discourse: The Next Steps

Under the precision medicine rubric, specifically computational psychiatry, objective individualized metrics of behaviors (biometrics) may be at the future juncture between physiological and psychological Autism research. However, current results that infer the dominance of Psychological constructs and tools driving the research arena raise fundamental questions as to the design, scope and precision of primary psychological methods—and their impact on Physiological assessment. While the current approach of co-keyword analysis limits the extent of this interpretation, it points toward this far-reaching and consistent psychological impact. As such, future studies of refined full-text analysis focused on methodologies may reveal a more nuanced understanding of the application of such tools and psychological methods across the Autism arena. Indeed, such investigation is warranted as current Psychological behavioral (observational) based inventories lack the precision (sensitivity and specificity), and adequate statistical framework (summative discrete scores, rather than continuous physiologically grounded metrics) to enable appropriate translation into the tenets of precision psychiatry (Torres et al., 2016; Torres and Whyatt, 2017). The implied existence of an implicit (and explicit) struggle between Psychological and Physiological metrics, combined with the apparent ‘driving’ force of Psychological constructs across the academic arena, may point toward an area of academic enquiry being constrained by Psychological tools and numeric scores that are not yet physiologically grounded, or may lack a scale derived from a properly defined metric space (Lord et al., 1989, 2000; Torres, 2018). With research scope inevitably impacting the direction of funding, and subsequent policy decisions that have a direct bearing on the lives of millions of Americans (and further afield), the persistent role (and adequacy) of Psychological constructs within the Physiological context must therefore be considered.

Future work utilizing similar types of analyses as those used in the present work, but using instead the peer-reviewed scientific literature from nations that provide universal medical insurance coverage, will give us a better sense of how differences in public health policy influence and restrict the type of autism-science we can afford to do in the United States.

## Author Contributions

CW conceived study, designed and created the analytical methods, implemented all code for analyses, processed all data, wrote and edited the paper. ET conceived study framework and designed platform for analyses, guided study, edited and revised paper. Both authors approved the last version of the manuscript.

## Conflict of Interest Statement

The authors declare that the research was conducted in the absence of any commercial or financial relationships that could be construed as a potential conflict of interest.

## Footnotes

1 Search completed August 2016.

2 This sampling size was selected based on the minimum yearly data length—i.e., 100 articles were extracted for the year with the fewest published articles (1994).

3 Note: betweenness centrality—another prominent measure of internal connectivity—was deemed inappropriate due to reliance on geodesic features.

4 https://www.autismspeaks.org/docs/sciencedocs/grants/treatment_rfa_2018.pdf

5 The ICD-10 is approved by experts from 193 member countries American Psychiatric Association (2009). ICD vs. DSM [Online]. Available: http://www.apa.org/monitor/2009/10/icd-dsm.aspx [Accessed], and came into widespread use in 1994.

6 For full diagnostic information for ASD according to the ICD-1O please see: http://apps.who.int/classifications/icd10/browse/2010/en#/F80-F89.

7 https://www.rxlist.com/risperdal-side-effects-drug-center.htm

## References

[B1] American Psychiatric Association (1994). *Diagnostic and Statistical Manual (DSM-IV).* Washington, DC: American Psychiatric Association.

[B2] American Psychiatric Association (2009). *Diagnostic and Statistical Manual (DSM-IV-TR).* Washington, DC: American Psychiatric Assocaition.

[B3] American Psychiatric Association (2013). *Diagnostic and Statistical Manual 5th Edition (DSM-5).* Washington, DC: American Psychiatric Association 10.1176/appi.books.9780890425596

[B4] Aria M. Cuccurullo C. (2017). Bibliometrix: an R-tool for comprehensive science mapping analysis. *J. Informetr.* 11 959–975. 10.1016/j.joi.2017.08.007

[B5] Asperger H. (1944). Die ‘Autistischen psychopathen’ im kindesalter. *Eur. Arch. Psychiatry Clin. Neurosci.* 117 76–136. 10.1007/BF01837709

[B6] Bonacich P. (1987). Power and centrality: a family of measures. *Am. J. Soc.* 92 1170–1182. 10.1086/228631

[B7] Callon M. Courtial J.-P. Laville F. (1991). Co-word analysis as a tool for describing the network of interactions between basic and technological research: the case of polymer chemsitry. *Scientometrics* 22 155–205. 10.1007/BF02019280

[B8] Chuang J. Gupta S. Manning C. Heer J. (2013). “Topic model diagnostics: assessing domain relevance via topical alignment,” in *Proceedings of the 30th International Conference on Machine Learning (ICML-13)*, (New York, NY: ACM),612–620.

[B9] R Core Team (2016). *R: A Language and Environment for Statistical Computing.* Vienna: R Foundation for Statistical Computing.

[B10] Cosgrove L. Bursztajn H. J. Krimsky S. (2009a). Developing unbiased diagnostic and treatment guidelines in psychiatry. *N. Engl. J. Med.* 360 2035–2036. 10.1056/NEJMc0810237 19420379

[B11] Cosgrove L. Bursztajn H. J. Krimsky S. Anaya M. Walker J. (2009b). Conflicts of interest and disclosure in the American Psychiatric Association’s Clinical Practice Guidelines. *Psychother. Psychosom.* 78 228–232. 10.1159/000214444 19401623

[B12] Cosgrove L. Krimsky S. (2012). A comparison of DSM-IV and DSM-5 panel members’ financial associations with industry: a pernicious problem persists. *PLoS Med.* 9:e1001190. 10.1371/journal.pmed.1001190 22427747 PMC3302834

[B13] Cosgrove L. Krimsky S. Vijayaraghavan M. Schneider L. (2006). Financial ties between DSM-IV panel members and the pharmaceutical industry. *Psychother. Psychosom.* 75 154–160. 10.1159/000091772 16636630

[B14] Cosgrove L. Krimsky S. Wheeler E. E. Kaitz J. Greenspan S. B. Dipentima N. L. (2014a). Tripartite conflicts of interest and high stakes patent extensions in the DSM-5. *Psychother. Psychosom.* 83 106–113. 10.1159/000357499 24458102

[B15] Cosgrove L. Shaughnessy A. F. Wheeler E. E. Krimsky S. Peters S. M. Freeman-Coppadge D. J. (2014b). From caveat emptor to caveat venditor: time to stop the influence of money on practice guideline development. *J. Eval. Clin. Pract.* 20 809–812. 10.1111/jep.12244 25327453

[B16] Coulter N. Monarch I. Konda S. Carr M. (1996). *An Evolutionary Perspective of Software Engineering Research Through Co-Word Analysis.* Pittsburgh, PA: Software Engineering Institute 10.21236/ADA310305

[B17] FDA (2006). *New Pediatric Labelling Information Database.* Available at: https://www.accessdata.fda.gov/scripts/sda/sdnavigation.cfm?sd=labelingdatabase&displayall=false&page=10

[B18] FDA (2007). *Risperdal Label.* Available at: https://www.accessdata.fda.gov/drugsatfda_docs/label/2007/020272s46s47,20588s36s37,21444s20s21lbl.pdf

[B19] Fombonne E. (2003). The prevalence of autism. *JAMA* 289 87–89. 10.1001/jama.289.1.87 12503982

[B20] Friston K. J. Stephan K. E. Montague R. Dolan R. J. (2014). Computational psychiatry: the brain as a phantastic organ. *Lancet Psychiatry* 1 148–158. 10.1016/S2215-0366(14)70275-5 26360579

[B21] Hill A. P. Zuckerman K. Fombonne E. (2015). “Epidemiology of autism spectrum disorders,” in *Translational Approaches to Autism Spectrum Disorder*, ed. de los Angeles Robinson-Agramonte M. (New York, NY: Springer), 13–38. 10.1007/978-3-319-16321-5_2

[B22] Hofmann S. G. (2014). Toward a cognitive-behavioral classification system for mental disorders. *Behav. Ther.* 45 576–587. 10.1016/j.beth.2014.03.001 24912469 PMC4234113

[B23] Insel T. R. (2009). Translating scientific opportunity into public health impact: a strategic plan for research on mental illness. *Arch. Gen. Psychiatry* 66 128–133. 10.1001/archgenpsychiatry.2008.540 19188534

[B24] Insel T. R. (2013). *Transforming Diagnosis*. Available at: https://www.nimh.nih.gov/about/directors/thomas-insel/blog/2013/transforming-diagnosis.shtml

[B25] Insel T. R. (2014). The NIMH research domain criteria (RDoC) project: precision medicine for psychiatry. *Am. J. Psychiatry* 171 395–397. 10.1176/appi.ajp.2014.14020138 24687194

[B26] Insel T. R. Cuthbert B. Garvey M. Heinssen R. Pine D. S. Quinn K. (2010). Research domain criteria (RDoC): toward a new classification framework for research on mental disorders. *Am. J. Psychiatry* 167 748–751. 10.1176/appi.ajp.2010.09091379 20595427

[B27] Insel T. R. Landis S. C. Collins F. S. (2013). Research priorities. The NIH BRAIN Initiative. *Science* 340 687–688. 10.1126/science.1239276 23661744 PMC5101945

[B28] Isenberg P. Isenberg T. Sedlmair M. Chen J. Möller T. (2017). Visualization as seen through its research paper keywords. *IEEE Trans. Vis. Comput. Graph.* 23 771–780. 10.1109/TVCG.2016.2598827 27875191

[B29] Kanner L. (1943). Autistic disturbances of affective contact. *Nerv. Child* 2 217–250. 4880460

[B30] Lord C. Dilavore P. C. Gotham K. (2012). *Autism Diagnostic Observation Schedule.* Torrance, CA: Western Psychological Services.

[B31] Lord C. Risi S. Lambrecht L. Cook E. H. Jr. Leventhal B. L. DiLavore P. C. (2000). The autism diagnostic observation schedule-generic: a standard measure of social and communication deficits associated with the spectrum of autism. *J. Autism Dev. Disord.* 30 205–223. 10.1023/A:1005592401947 11055457

[B32] Lord C. Rutter M. Goode S. Heemsbergen J. Jordan H. Mawhood L. (1989). Autism diagnostic observation schedule: a standardized observation of communicative and social behavior. *J. Autism Dev. Disord.* 19 185–212. 10.1007/BF02211841 2745388

[B33] Matson J. L. Kozlowski A. M. (2011). The increasing prevalence of autism spectrum disorders. *Res. Autism Spectr. Disord.* 5 418–425. 10.1016/j.rasd.2010.06.004

[B34] McCracken J. T. Mcgough J. Shah B. Cronin P. Hong D. Aman M. G. (2002). Risperidone in children with autism and serious behavioral problems. *N. Engl. J. Med.* 347 314–321. 10.1056/NEJMoa013171 12151468

[B35] Newman M. E. (2006). Modularity and community structure in networks. *Proc. Natl. Acad. Sci. U.S.A.* 103 8577–8582. 10.1073/pnas.0601602103 16723398 PMC1482622

[B36] Nordin V. Gillberg C. (1996). Autism spectrum disorders in children with physical or mental disability or both. I: clinical and epidemiological aspects. *Dev. Med. Child Neurol.* 38 297–313. 10.1111/j.1469-8749.1996.tb12096.x 8641535

[B37] Office of Autism Research Coordination (OARC) and and National Institute of Mental Health and Thomson Reuters Inc. (2012). *Autism Spectrum Disorder Research Publications Analysis Report: The Global Landscape of Autism Reseach.* Available at: http://iacc.hhs.gov/publications-analysis/july2012/index.shtml

[B38] Okubo Y. (1997). *Bibliometric Indicators and analysis of Research Systems.* Paris: OECD 10.1787/208277770603

[B39] Reichardt J. Bornholdt S. (2006). Statistical mechanics of community detection. *Phys. Rev. E* 74:016110. 10.1103/PhysRevE.74.016110 16907154

[B40] Singh J. (2011). “The vanishing diagnosis of Asperger’s disorder,” in *Sociology of Diagnosis*, eds McGann P.J. Hutson D. J. (Bingley: Emerald Group Publishing), 235–257.

[B41] Singh J. Hallmayer J. Illes J. (2007). Interacting and paradoxical forces in neuroscience and society. *Nat. Rev. Neurosci.* 8 153–160. 10.1038/nrn2073 17237806 PMC1885680

[B42] Sparrow S. S. Balla D. A. Cicchetti D. V. Harrison P. L. Doll E. A. (1984). *Vineland Adaptive Behavior Scales.* Circle Pines, MN: American Guidance Service.

[B43] Topalli M. Ivanaj S. (2016). Mapping the evolution of the impact of economic transition on Central and Eastern European enterprises: a co-word analysis. *J. World Bus.* 51 744–759. 10.1016/j.jwb.2016.06.003

[B44] Torres E. B. (2018). *Objective Biometric Methods for the Diagnosis and Treatment of Nervous System Disorders.* New York, NY: Elsevier.

[B45] Torres E. B. Denisova K. (2016). Motor noise is rich signal in autism research and pharmacological treatments. *Sci. Rep.* 6:37422. 10.1038/srep37422 27869148 PMC5116649

[B46] Torres E. B. Isenhower R. W. Nguyen J. Whyatt C. Nurnberger J. I. Jose J. V. (2016). Toward precision psychiatry: statistical platform for the personalized characterization of natural behaviors. *Front. Neurol.* 7:8. 10.3389/fneur.2016.00008 26869988 PMC4735831

[B47] Torres E. B. Mistry S. Caballero C. Whyatt C. P. (2017). Stochastic signatures of involuntary head micro-movements can be used to classify females of ABIDE into different subtypes of neurodevelopmental disorders. *Front. Integr. Neurosci.* 11:10. 10.3389/fnint.2017.00010 28638324 PMC5461345

[B48] Torres E. B. Whyatt C. (2017). *Autism: the Movement Sensing Perspective.* Boca Raton, FL: CRC Press 10.1201/9781315372518

[B49] U.S. Department of Justice (2013a). *Filed Documentation.* Available at: https://www.justice.gov/sites/default/files/usao-edpa/legacy/2014/02/11/JanssenPharma_information.pdf [accessed 7 August, 2017].

[B50] U.S. Department of Justice (2013b). *Johnson & Johnson to Pay More Than $2.2 Billion to Resolve Criminal and Civil Investigations.* Available at: https://www.justice.gov/opa/pr/johnson-johnson-pay-more-22-billion-resolve-criminal-and-civil-investigations [accessed 6 August, 2017].

[B51] Wang X.-J. Krystal J. H. (2014). Computational psychiatry. *Neuron* 84 638–654. 10.1016/j.neuron.2014.10.018 25442941 PMC4255477

[B52] Watts G. (2012). Critics attack DSM-5 for overmedicalising normal human behaviour. *BMJ* 344:e1020. 10.1136/bmj.e1020 22327355

[B53] Williams R. Runco M. A. Berlow E. (2016). Mapping the themes, impact, and cohesion of creativity research over the last 25 years. *Creat. Res. J.* 28 385–394. 10.1080/10400419.2016.1230358

[B54] Wing L. (1981). Asperger’s syndrome: a clinical account. *Psychol. Med.* 11 115–129. 10.1017/S0033291700053332 7208735

[B55] Wing L. (1997). The autistic spectrum. *Lancet* 350 1761–1766. 10.1016/S0140-6736(97)09218-0 9413479

[B56] World Health Organization (1994). *The ICD-10 Classification of Mental and Behavioural Disorders: Clinical Descriptions and Diagnostic Guidelines.* Geneva: World Health Organization.

[B57] World Health Organization (2016). *The International Statistical Classification of Dieases and Related Health Problems 10th Revision (ICD-10).* Geneva: World Health Organization.

[B58] Zhang W. Zhang Q. Yu B. Zhao L. (2015). Knowledge map of creativity research based on keywords network and co-word analysis, 1992–2011. *Qual. Quant.* 49 1023–1038. 10.1007/s11135-014-0032-9

