# Supplementary material for: Autism Research: An Objective Quantitative Review of Progress and Focus Between 1994 and 2015
Source: Front Psychol. 2018 Aug 23;9:1526. doi: 10.3389/fpsyg.2018.01526 (PMC6116169; doi:10.3389/fpsyg.2018.01526)
Supplement: Supplementary file 1 [file Table_1.DOCX]

| \| **Rank** \| **Publication: Author (Year), Brief Description** \| **Classification** \| \| --- \| --- \| --- \| \| 1 \| American Psychiatric Association (2006). *Publication of the DSM-IV-TR.* \| Psychology \| \| 2 \| Lord, Rutter, and Le Couteur (1994). *Publication of the Autism Diagnostic Observation Schedule.* \| Psychology \| \| 3 \| American Psychiatric Association (1994). *Publication of the DSM-IV.* \| Psychology \| \| 4 \| Lord, Risi, Lambrecht, Cook, Leventhal, DiLavore, Pickles, Rutter (2000). *Publication of the Autism Diagnostic Observation Schedule – 2^nd^ Edition.* \| Psychology \| \| 5 \| Kanner (1943). *Seminal text describing behavioral observations of Autism.* \| Psychology \| \| 6 \| American Psychiatric Association (2013). *Publication of the DSM-5.* \| Psychology \| \| 7 \| Bailey, Le Couteur, Gottesman, Bolton, Simonoff, Yuzda Andm, Rutter (1995). *British twin study illustrating potential genetic etiology of ASD.* \| Physiology \| \| 8 \| Sparrow, Balla, Cicchetti, Harrison, and Doll (1984). *Publication of the Vineland adaptive behavior scales.* \| Psychology \| \| 9 \| Mullen (1995). *Publication of the Mullen Scales of early learning.* \| Psychology \| \| 10 \| Lord, Rutter, Le Couteur (1994). *Publication of the Autism Diagnostic Interview-Revised.* \| Psychology \| \| 11 \| Lord, Rutter, Goode, Heemsbergen, Jordan, Mawhood, Schopler (1989). *Precursor publication of the Autism Diagnostic Observation Schedule.* \| Psychology \| |
| --- | --- | --- | --- | --- | --- | --- | --- | --- | --- | --- | --- | --- | --- | --- | --- | --- | --- | --- | --- | --- | --- | --- | --- | --- | --- | --- | --- | --- | --- | --- | --- | --- | --- | --- | --- | --- |
| **Supplementary Table 1** Summary of top cited articles extracted from the text corpus across the period of 1994-2015. Reflecting the overarching key-word analysis, and classification analysis, the core citations show a disposition toward publications within the arena of Psychology and Psychiatry. Furthermore, such citations include – perhaps unsurprisingly – diagnostic criteria and clinical tools |
