# Supplementary material for: Autism Research: An Objective Quantitative Review of Progress and Focus Between 1994 and 2015
Source: Front Psychol. 2018 Aug 23;9:1526. doi: 10.3389/fpsyg.2018.01526 (PMC6116169; doi:10.3389/fpsyg.2018.01526)
Supplement: Supplementary file 2 [file Table_2.DOCX]

| \| **Rank** \| **Publication: Author (Year), Brief Description** \| **Classification** \| \| --- \| --- \| --- \| \| 1 \| American Psychiatric Association (1994). *Publication of the DSM-IV.* \| Psychology \| \| 2 \| Lord, Rutter, and Le Couteur (1994). *Publication of the Autism Diagnostic Observation Schedule.* \| Psychology \| \| 3 \| Kanner (1943). *Seminal text describing behavioral observations of Autism.* \| Psychology \| \| 4 \| American Psychiatric Association (1987) *Publication of the DSM-III-R* \| Psychology \| \| 5 \| Bailey, Le Couteur, Gottesman, Bolton, Simonoff, Yuzda Andm, Rutter (1995). *British twin study illustrating potential genetic etiology of ASD.* \| Physiology \| \| 6 \| Sparrow, Balla, Cicchetti, Harrison, and Doll (1984). *Publication of the Vineland adaptive behavior scales.* \| Psychology \| \| 7 \| Lord, Rutter, Goode, Heemsbergen, Jordan, Mawhood, Schopler (1989). *Precursor publication of the Autism Diagnostic Observation Schedule.* \| Psychology \| \| 8 \| Le Couteur, Rutter, Lord, Rios, Robertson, Holdgrafer, McLennan (1989) *Autism Diagnostic interview: a standardized investigator based instrument.* \| Psychology \| \| 9 \| Baron-Cohen, Leslie, Frith (1985) *Discussion regarding lack of theory of mind associated with ASD.* \| Psychology \| \| 10 \| Lovaas (1987) *Presentation of behavioral intervention – precursor to Applied Behavioral Analysis techniques.* \| Psychology \| |
| --- | --- | --- | --- | --- | --- | --- | --- | --- | --- | --- | --- | --- | --- | --- | --- | --- | --- | --- | --- | --- | --- | --- | --- | --- | --- | --- | --- | --- | --- | --- | --- | --- | --- |
| **Supplementary Table 2** Summary of top cited articles extracted from the text corpus across the period of 1994-2004 – Decade 1. Reflecting the overarching key-word analysis, and classification analysis for this timeframe, the core citations show a disposition toward publications within the arena of Psychology and Psychiatry |
