# Supplementary material for: Autism Research: An Objective Quantitative Review of Progress and Focus Between 1994 and 2015
Source: Front Psychol. 2018 Aug 23;9:1526. doi: 10.3389/fpsyg.2018.01526 (PMC6116169; doi:10.3389/fpsyg.2018.01526)
Supplement: Supplementary file 4 [file Table_4.DOCX]

| **Summary normalized connectivity metrics** | | | | | | |  |
| --- | --- | --- | --- | --- | --- | --- | --- |
| **Level of analysis** | **Full Corpus** | | **Decade 1** | | **Decade 2** | |  |
|  | *External* | *Internal* | *External* | *Internal* | *External* | *Internal* |  |
| ***Full*** | 169602 | 334.05050 | 10786 | 27.6864 | 17917 | 31.8541 |  |
| ***Refined*** | 71079.500 | 129.0067 | 4907 | 18.6880 | 19252 | 39.8241 |  |
| **Supplementary Table 4** Summary of normalized median external and internal connectivity metrics for each level of analysis: Full corpus, Decade 1 and Decade 2. As demonstrated, removal of core network hubs and assessment of this refined network leads to an intuitive reduction in both internal and external connectivity across both the Full corpus and Decade 1. In contrast, Decade 2 demonstrates an increase in levels of connectivity—an increase that is perhaps reflected in the consolidation of underlying network modularity; in contrast to previous levels of analysis | | | | | | | |
