# Supplementary material for: Autism Research: An Objective Quantitative Review of Progress and Focus Between 1994 and 2015
Source: Front Psychol. 2018 Aug 23;9:1526. doi: 10.3389/fpsyg.2018.01526 (PMC6116169; doi:10.3389/fpsyg.2018.01526)
Supplement: Supplementary file 5 [file Table_5.DOCX]

|  | | | **Summary Modular Characteristics** | | | | | | |
| --- | --- | --- | --- | --- | --- | --- | --- | --- | --- |
|  | | | **Module 1** | **Module 2** | **Module 3** | **Module 4** | **Module 5** | **Module 6** | **Module 7** |
| **Full Corpus Analysis** | **Full Corpus** | *Thematic classification* | Psychological | Physiological | - | - | - | - | - |
|  |  | *Number of Keywords* | 760 | 1000 | - | - | - | - | - |
|  |  | *Maximally Interconnected Nodes* | Spectrum & Children | Mental & Autism | - | - | - | - | - |
|  | **Decade 1** | *Thematic classification* | Physiological | Psychological | Physiological | Psychological | Psychological | - | - |
|  |  | *Number of Keywords* | 321 | 389 | 110 | 279 | 95 | - | - |
|  |  | *Maximally Interconnected Nodes* | Individual & Children | Infant & Brain | Linkage & Disorder | Behavior & Autism | Mental & Adults | - | - |
|  | **Decade 2** | *Thematic classification* | Psychological | Physiological | Physiological | Physiological | - | - | - |
|  |  | *Number of Keywords* | 664 | 520 | 277 | 107 | - | - | - |
|  |  | *Maximally Interconnected Nodes* | Spectrum & Children | Schizophrenia & Autism | Cerebral & Brain | Pregnant & Maternal | - | - | - |
| **Trimmed Corpus Analysis** | **Full Corpus** | *Thematic classification* | Psychological | Physiological | Physiological | Physiological | - | - | - |
|  |  | *Number of Keywords* | 745 | 472 | 306 | 225 | - | - | - |
|  |  | *Maximally Interconnected Nodes* | Young- & Behavior | Synaptic & Mouse | Linkage & Genome | Brain & Abnormalities | - | - | - |
|  | **Decade 1** | *Thematic classification* | Physiological | Psychological | Physiological | Psychological | Psychological | Physiological | Physiological |
|  |  | *Number of Keywords* | 354 | 217 | 251 | 174 | 115 | 73 | 53 |
|  |  | *Maximally Interconnected Nodes* | Linkage & Disorder | Young- & Behavior | Infant & Brain | Pervasive & Individual | Mind & Deficits | MMR | Placebo & Double |
|  | **Decade 2** | *Thematic classification* | Physiological | Psychological | Psychological | - | - | - | - |
|  |  | *Number of Keywords* | 898 | 390 | 270 | - | - | - | - |
|  |  | *Maximally Interconnected Nodes* | Genome & De-Novo | Young- & Behavior | Deficits & Attention | - | - | - | - |
| **Supplementary Table 5** Summary of modular characteristics, including maximally interconnected nodes per module, at each primary level of analysis. Note: The single terms Over-S (subsuming the following terms: ‘Over-selected stimuli’ and ‘ Over-selectivity’) and Chain (subsuming the following terms: ‘Chain’ and ‘Chain Fatty-acids’) with minimal connections were removed from reporting for trimmed Decade1 | | | | | | | | | |
