# Supplementary material for: Autism Research: An Objective Quantitative Review of Progress and Focus Between 1994 and 2015
Source: Front Psychol. 2018 Aug 23;9:1526. doi: 10.3389/fpsyg.2018.01526 (PMC6116169; doi:10.3389/fpsyg.2018.01526)
Supplement: Supplementary file 6 [file Data_Sheet_1.DOCX]

**Online Supplementary Material**

**Online Supplementary Section 1: Maximally Occurring Keywords**

Adolescence, Adulthood, Adults, Age, Asperger’s, Association, Attention, Autism, Behavior, Brain, Cerebral, Child, Childhood, Children, Cognition, Communication, De-Novo, Deficiency, Development, Diagnosis, Disability, Disease, Disorder, Down-Syndrome, Early, Emotion, Expression, Familial, Family, Follow, Fragile-X, Functioning, Gene, Genome, Health, High-functioning, Human, Identification, Impairment, Individual, Infant, Intelligence, Intervention, Joint-Attention, Language, Linkage, Me, Men, Mental, Mice, Mind, Model, Mother, Mouse, Mutation, Neural, Parent, Perception, Performance, Pervasive, Phenotype, Popular, Pre-school, Prevalence, Protein, Psychiatric, Psychological, Rat, Receptor, Recognition, Response, Rick, Schizophrenia, School, Self, Skill, Social, Spectrum, Speech, Student, Symptoms, Young, Young-Adults.

**Online Supplementary Section 2: Keywords for Thematic Module Classification**

**Overarching Corpus: Psychological Module (M1).** Children, Spectrum, Pervasive, Behavior, Young-adults, Asperger’s, Individual, Diagnosis, Infant, Adolescence, Social, High-Functioning, Development, Intervention, Language, Communication, Deficit, Prevalence, Disability, Childhood, Adults, Pre-school, Parent, Attention, Intelligence, Age, Joint-Attention, Symptoms, Psychological, Early, Skills, Perception, Functioning, Psychiatry, Population, Follow-up, Cognition, Recognition, Mind, Health, Family, Student, Toddler, Down-syndrome, Speech, Validity, Emotion, Mother, School, Executive, Repetition, Response, Performance, Stress, Reliability, Familiar, Generation, Pattern, Anxiety, Epidemiology, Double, Facial, Challenges, Obsessive, Self-Injurious, Instrument, Meta-analysis, Acquisition, Motor, Depression, Outcome, Risk-Factors, Autistic, Working. **Physiological Module (M2).** Autism, Mental, Disorder, Brain, Schizophrenia, Human, Expression, Mouse, Association, Mutation, De-Novo, Genome, Fragile-X, Gene, Phenotype, Cerebral, Protein, Disease, Identification, Neuron, Reception, Risk, Visual, Mice, Chromosome, Neural, Bi-Polar, Synaptic, Genetic, Micro, Systems, Deletion, Linkage, Epilepsy, Maternal, Abnormal, Activation, Copy Number, Completion, Model, Cortical, Cortex, Variant, Long-Term, Pre-frontal, Mechanism, Rett-Syndrome, Messenger, Hippocampus, Differential, White-Matter, Transcription, Memory, Methylene, Alzheimer’s, Amygdala, Inhibition, Gene-Expression, Structure, Genes, Multiplication, Prenatal, Duplication, Mirror, Animal, Males, In-Vivo, Molecular, Binding, Serotonin, Williams-Syndrome, Temporal, Sex-differences, MRI, Neuropathy, Increase, Humans, Major, Network, Inbred, Corpus, Neuron, Frontal, Angelman Syndrome, Growth, Environment, Polymorph, Dendrite, Dysfunction, Candidate, Central NS, Alpha-waves, Glutamine, FMRI.

**1994-2005: Physiological Module (M1).** Disorder, Linkage, Chromosome, Genomic, Association, Serotonin, Susceptible, Expression, Gene, Twin, Spectrum, Genetics, Disease, Genome, Region, Receptor, Multiple, Angelman-Syndrome, Proximal, 15Q11-, Complete, Identification, Epilepsy, Protein, Population, Duplicate, Variants, Pairs, Etiology, Marker, Heterogeneous, Mutation, Exposure, Candidate-genes, Locus, Pedigree. **Psychological Module (M2).** Behavior, Autism, Language, Young-adults, Preschool, Intervention, Skills, Disabilities, Communication, Deficits, Social, Development, Joint-Attention, Mind, Student, Early, Acquisition, Speech, Non-verbal, Attention, Normal, Mother, Severity, Impairment, Imitation, Response, Play, Emotion, Executive, Reinforcement, Profile, Program. **Physiological Module (M3)**. Infant, Brain, Abnormalities, Schizophrenia, Cerebellum, Posterior, Cerebral, Human, Position, Recognition, Corpus, Hippocampus, Cortex, Size, Head-Circumference, MRI, Temporal, Frontal, Visual, Activation, Maternal, Perception, Neuropathy, Neuron, Neural, Facial. **Psychological Module (M4).** Children, Pervasive, Individual, Diagnosis, Children, Asperger’s, Family, Follow-up, Parent, Prevalence, Age. **Physiological Module (M5)**. Mental, Adults, Double, Adolescent, Autistic, Placebo, Obsessive, Psychological.

**2006-2015: Psychological Module (M1).** Children, Spectrum, Young-, Behavior, Individual, Asperger’s, Diagnosis, Pervasive, Adolescent, High-Functioning, Social, Development, Intervention, Communication, Disability, Language, Prevalence, Adults, Attention, Deficit, Infant, Psychology, Childhood, Parent, Preschool, Age, Symptoms. **Physiological Module (M2).** Autism, Mental, Schizophrenia, Disorder, Mouse, De-novo, Expression, Association, Fragile (X), Genome, Phenotype, Neuron, Risk, Mutation, Identification, Gene, Chromosome, Family, Bi-polar, Synaptic, Model. **Physiological Module (M3).** Brain, Human, Function, Cerebral, Cortical, Facial, FMRI, Neural, White matter, MRI, Prefrontal. **Physiological Module (M4).** Maternal, Pregnant, Exposure, Perinatal.

**(Insert Supplementary Fig.1 about here)**

**Supplementary Fig.1** Illustrative example of k-means clustering to isolate the top journals of publication for categorization per five-year period. **(a)** Summary of number publications and associated journal ranking across each year. Note – mean values from across each five-year period is denoted. The Journal of Autism and Developmental Disorders emerged as the highest ranking journal of publication within the field of ‘Autism’ across all years—1994 to 2015; a journal that was categorized as ‘interdisciplinary’ in its approach. K-means clustering was performed (see **b for illustrative example of 1994**) per year, identifying the *top* ranked journals (those with high frequency metrics). Those groups prior to the k-means identified demarcation of tail data (**c**) were extracted for subsequent categorization (see main text). In this example (1994), the top 9 ranked journals are extracted for further consideration [https://figshare.com/s/728b969af4ee7ba5247d](https://figshare.com/s/728b969af4ee7ba5247d)

**(Insert Supplementary Fig.2 about here)**

**Supplementary Fig.2** Timeline of maximal and minimal nodes across the corpus. In line with the overarching growth in publication rate, the number of maximal nodes across the timeframe displays a steady increase <https://figshare.com/s/771d6535f64f748ed771>

**(Insert Supplementary Fig.3 about here)**

**Supplementary Fig.3** Summary of co-keyword analysis completed across the key years of analysis. Start year (1994), isolated year of publication increase i.e. Inflection year (2006), and End year (2015). Keyword co-occurrence matrices are visualized using graph theory methods, and modularity analytics applied to identify self-clustering networks (**a**). To aid accessibility, these modules are visualized as colored concentric rings, each reflecting a modules singular rank and theme (**Psychological Blue, Physiological Red, Interdisciplinary Green – b**). Node size reflects normalized Eigenvector centrality, with prominent internal nodes labeled (see **Online Supplementary Section 1** **for full prominent node listing**). Modular internal and external connectivity metrics were extracted, normalized and visualized relative to normalized corpus median values (**c**). Unfortunately, due to the highly disparate landscape of the start year (1994), modularity analytics are not presented. However, as illustrated across the inflection year—end year, the underlying strength and dominance of psychological self-clustering modules evolves <https://figshare.com/s/68ae90cd84ce83742b92>

**(Insert Supplementary Fig.4 about here)**

**Supplementary Fig.4** Summary matrices visualizing the external connections between modules across both the inflection year (2006) and end year (2015). Note: these are examined at the full corpus level, and display the continued role of psychological terminology to create core areas of thematic cross talk for the physiological clusters. Of interest, the variability and range of external thematic interconnections evolves across this level of analysis, with the prominent psychological module of each year (M1), while still dominant via modularity ranking, displaying a more nuanced and refined level of exchange by 2015. Colors are indicative of normalized levels of external connectivity, with lighter colors denoting a larger number of normalized connections (note: original count data is clearly indicated). Prominent internal module nodes (keywords) are indicated on the x-axis, while core external partner nodes are represented on the y-axis [https://figshare.com/s/06348d92942c66fda20c](https://figshare.com/s/06348d92942c66fda20c)
