# Supplementary figures and images for: Autism Research: An Objective Quantitative Review of Progress and Focus Between 1994 and 2015
Source: Front Psychol. 2018 Aug 23;9:1526. doi: 10.3389/fpsyg.2018.01526 (PMC6116169; doi:10.3389/fpsyg.2018.01526)

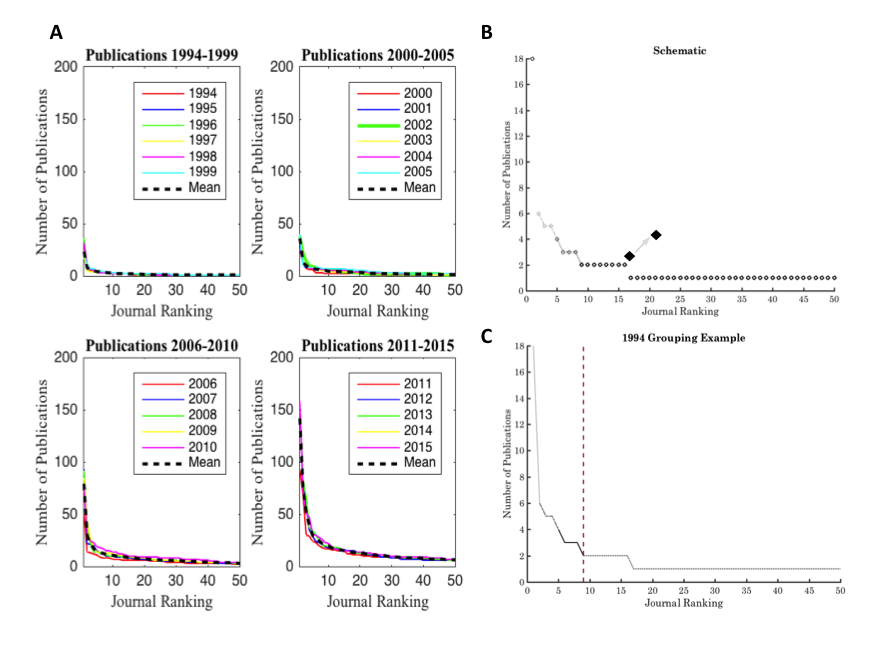

Supplement: Supplementary file 7 [file Image_1.tiff]

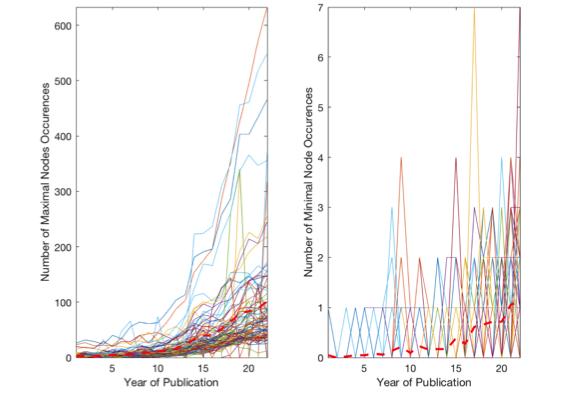

Supplement: Supplementary file 8 [file Image_2.tiff]

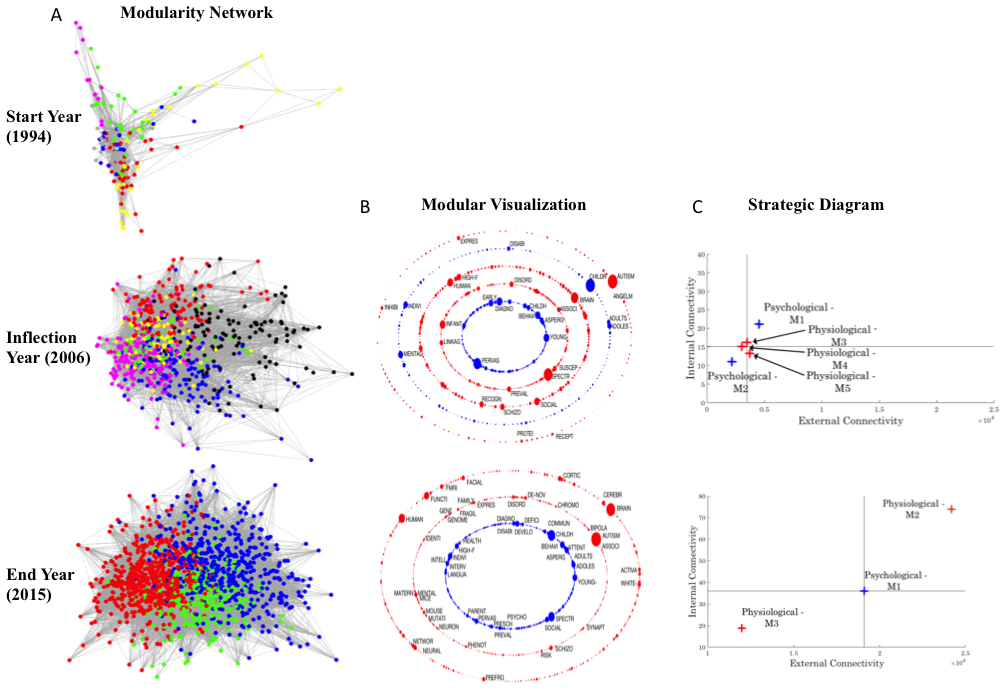

Supplement: Supplementary file 9 [file Image_3.tiff]

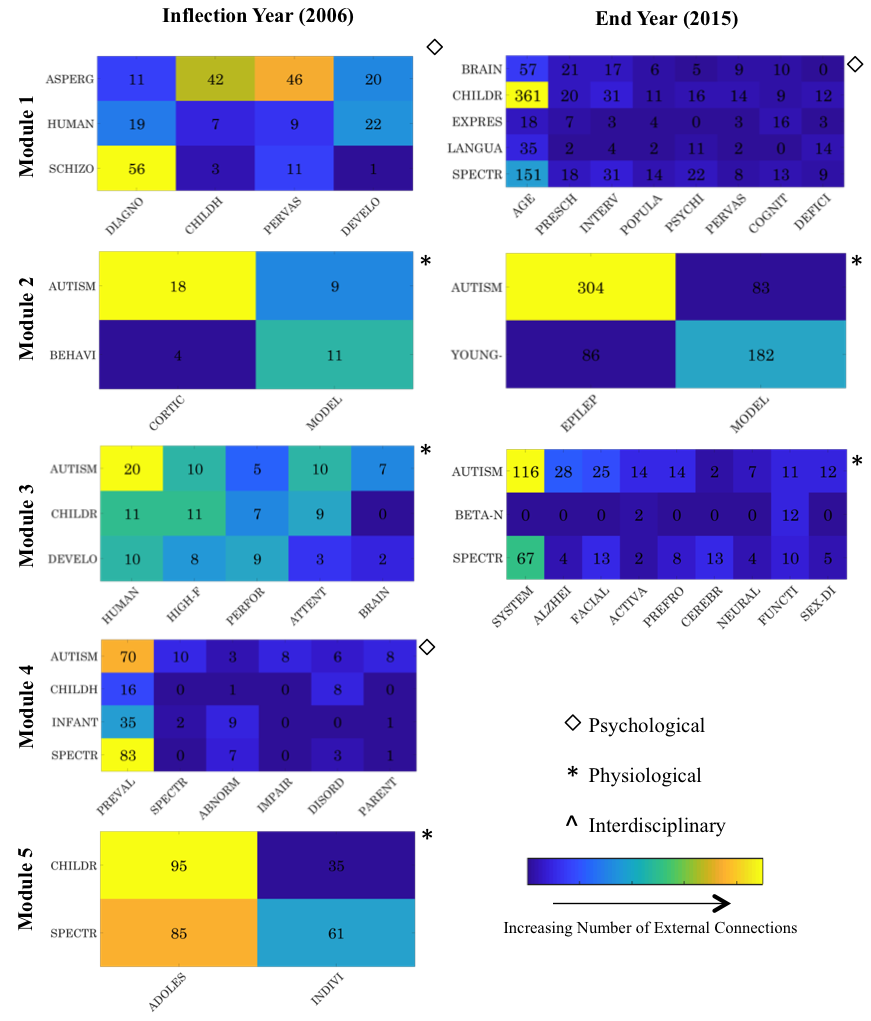

Supplement: Supplementary file 10 [file Image_4.tiff]
